# Radioproteomics stratifies molecular response to antifibrotic treatment in pulmonary fibrosis

**DOI:** 10.1172/jci.insight.181757

**Published:** 2024-07-16

**Authors:** David Lauer, Cheryl Y. Magnin, Luca R. Kolly, Huijuan Wang, Matthias Brunner, Mamta Chabria, Grazia M. Cereghetti, Hubert S. Gabryś, Stephanie Tanadini-Lang, Anne-Christine Uldry, Manfred Heller, Stijn E. Verleden, Kerstin Klein, Adela-Cristina Sarbu, Manuela Funke-Chambour, Lukas Ebner, Oliver Distler, Britta Maurer, Janine Gote-Schniering

**Affiliations:** 1 Department of Rheumatology and Immunology, Inselspital, Bern University Hospital, and; 2 Lung Precision Medicine (LPM), Department for BioMedical Research (DBMR), University of Bern, Bern, Switzerland.; 3 Department of Rheumatology, Center of Experimental Rheumatology, University Hospital Zurich, University of Zurich, Zurich, Switzerland.; 4 Graduate School for Cellular and Biomedical Sciences, University of Bern, Switzerland.; 5 Department of Health Sciences and Technology, ETH Zurich, Zurich, Switzerland.; 6 Department of Diagnostic, Interventional, and Pediatric Radiology, Inselspital, Bern University Hospital, University of Bern, Bern, Switzerland.; 7 Department of Radiation Oncology, University Hospital Zurich, Zurich, Switzerland.; 8 Proteomics & Mass Spectrometry Core Facility (PMSCF), DBMR, University of Bern, Bern, Switzerland.; 9 Department of ASTARC, University of Antwerp, Antwerp, Wilrijk, Belgium.; 10 Department of Pulmonary Medicine, Allergology and Clinical Immunology, Inselspital, Bern University Hospital, University of Bern, Bern, Switzerland.; 11 Department of Radiology, Cantonal Hospital Lucerne, Luzern, Switzerland.; 12 Institute for Radiology, Hirslanden Bern Klinik Beau-Site, Bern, Switzerland.

**Keywords:** Pulmonology, Therapeutics, Diagnostic imaging, Fibrosis, Proteomics

## Abstract

Antifibrotic therapy with nintedanib is the clinical mainstay in the treatment of progressive fibrosing interstitial lung disease (ILD). High-dimensional medical image analysis, known as radiomics, provides quantitative insights into organ-scale pathophysiology, generating digital disease fingerprints. Here, we performed an integrative analysis of radiomic and proteomic profiles (radioproteomics) to assess whether changes in radiomic signatures can stratify the degree of antifibrotic response to nintedanib in (experimental) fibrosing ILD. Unsupervised clustering of delta radiomic profiles revealed 2 distinct imaging phenotypes in mice treated with nintedanib, contrary to conventional densitometry readouts, which showed a more uniform response. Integrative analysis of delta radiomics and proteomics demonstrated that these phenotypes reflected different treatment response states, as further evidenced on transcriptional and cellular levels. Importantly, radioproteomics signatures paralleled disease- and drug-related biological pathway activity with high specificity, including extracellular matrix (ECM) remodeling, cell cycle activity, wound healing, and metabolic activity. Evaluation of the preclinical molecular response–defining features, particularly those linked to ECM remodeling, in a cohort of nintedanib-treated fibrosing patients with ILD, accurately stratified patients based on their extent of lung function decline. In conclusion, delta radiomics has great potential to serve as a noninvasive and readily accessible surrogate of molecular response phenotypes in fibrosing ILD. This could pave the way for personalized treatment strategies and improved patient outcomes.

## Abstract 2

Computed tomography–derived imaging feature profiles, known as radiomics, act as a surrogate of molecular response to antifibrotic treatment in experimental lung fibrosis.

## Introduction

Fibrotic remodeling of the lung interstitium is the shared pathomechanism across various interstitial lung diseases (ILDs) of different etiologies, including idiopathic pulmonary fibrosis (IPF) and connective tissue disease–associated (CTD-associated) ILD as the most prevalent subtypes. For patients with IPF and other ILDs with a progressive fibrosing (PF-ILD) phenotype, treatment with the antifibrotic multitarget tyrosine kinase inhibitor nintedanib is recommended (1, 2). While nintedanib has proven effective in slowing pulmonary function decline in multiple clinical trials, it comes with a relatively high rate of side effects (3–5). Consequently, there is a pressing need to assess treatment efficacy and identify individuals who may not benefit from therapy early in the disease course.

Current evaluation of treatment response primarily relies on longitudinal lung function measurements, which are prone to intrapatient variability, can be influenced by extrapulmonary parameters and lack insights into the underlying molecular response (6, 7). Liquid- or tissue-derived readouts could partially address these limitations, but validated biomarkers are not yet available and repeated lung biopsies are not a viable option due to the associated interventional risks (8). Radiomics analysis of routinely performed high-resolution computed tomography (HRCT) scans has great potential to serve as a noninvasive solution for evaluation of treatment response in individual patients in 4 dimensions (3D space + time) (9, 10). Radiomic features are computationally retrieved and quantitative data extracted from radiological imaging data, which describe the tissue in terms of its intensity, texture, and shape properties (11), thus creating digital disease fingerprints (12). The added value compared with conventional visual radiological analysis or quantitative characterization methodologies, such as CALIPER (13), lies in their ability to capture image phenotypes beyond human visual perception (14), thereby aiming to close the gap between patient screening and precision medicine (15).

Radiomics is based on the premise that the underlying pathophysiology is reflected in the imaging phenotype and that radiomic features can quantify these links, offering insights into organ-scale pathophysiology. Previous work including our own has shown that radiomics can convey morphologic and molecular tissue characteristics with important implications for personalized diagnosis and prognostication (16–19). Delta radiomics, which quantifies the feature variation between 2 imaging time points and, thus, captures longitudinal phenotypic changes, has emerged as a method to predict and quantify treatment response in various types of cancer (20–22). Its potential for the stratification of antifibrotic treatment response in (progressive) fibrosing ILD remains to be studied.

This study aimed to evaluate whether delta radiomics can be used to stratify the degree of molecular response to antifibrotic treatment with nintedanib using the well-established bleomycin-induced lung fibrosis model.

## Results

### Delta radiomics uncovers heterogeneity in antifibrotic drug response.

**Figure 1:**
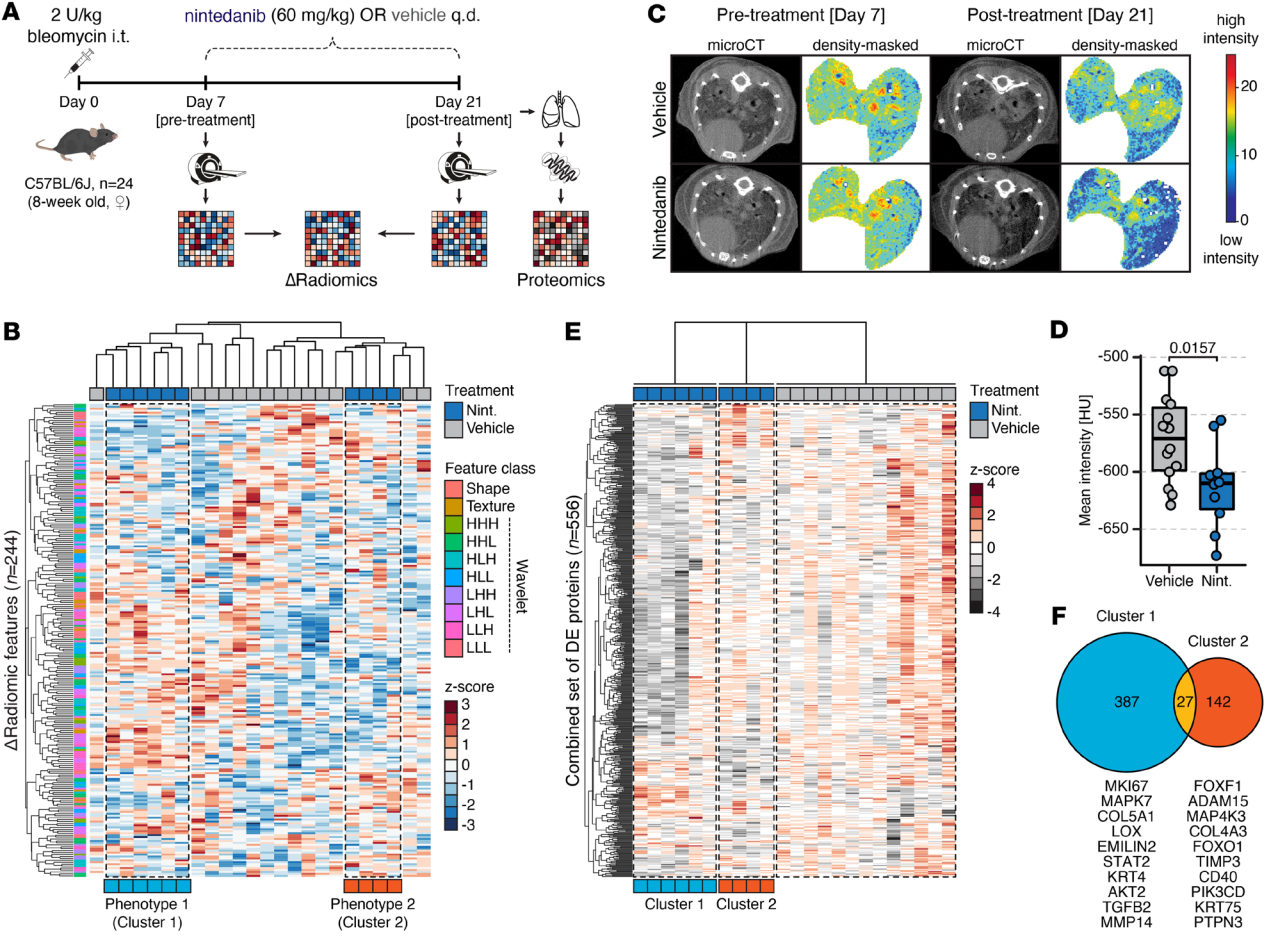
Delta radiomics uncovers heterogeneity in antifibrotic drug response. (**A**) Experiment schematic. C57BL/6J mice with bleomycin-induced lung fibrosis received treatment with nintedanib (*n* = 10) or vehicle (*n* = 14). Lung μCT scans were acquired of each mouse before (day 7) and after treatment (day 21) for analysis of radiomic measures. The change in radiomic feature expression was expressed as delta radiomics. Lung tissue was collected 24 hours after the final treatment application for molecular analyses. (**B**) Heatmap displaying the results of unsupervised hierarchical clustering of *Z*-scored delta radiomic features (*n* = 244) in all mice. Treatment groups and the class of each delta radiomic variable are indicated. (**C**) Representative lung μCT images and matching density-masked lobes of nintedanib- and vehicle-treated mice with bleomycin-induced lung fibrosis at pre- and posttreatment levels. (**D**) Lung tissue density expressed as mean Hounsfield unit (HU) intensity after treatment. Unpaired Student’s *t* test was used to compare the groups. (**E**) Heatmap showing the expression profiles of the combined set of DE proteins (*n* = 556) in cluster 1 and cluster 2 compared with vehicle-treated mice. Log_2_-transformed protein expression values were *Z*-scored. (**F**) Venn diagram depicting the number of differentially expressed proteins in cluster 1 (*n* = 414) and cluster 2 (*n* = 169) compared with the vehicle group. Selected DE proteins unique to cluster 1 or cluster 2 with functions implicated in disease pathophysiology are denoted.

To study the effects of antifibrotic treatment on radiomic signatures, we collected μCT-derived radiomic features in mice with bleomycin-induced lung fibrosis (*n* = 24) before (day 7) and after (day 21) treatment with nintedanib (*n* = 10) or vehicle (*n* = 14) (Figure 1A). The change in feature expression between pre- and posttreatment stages was quantified as delta radiomics. We considered only variables that were stable (intraclass correlation coefficient [ICC] ≥ 0.75) against semiautomatic lung segmentation and excluded highly correlated (Spearman’s ρ ≥ 0.85) features, resulting in a final set of 244 delta radiomic features that entered analysis (Supplemental Figure 1, A–C; supplemental material available online with this article; https://doi.org/10.1172/jci.insight.181757DS1).

Unsupervised hierarchical clustering of delta radiomics revealed heterogeneous response profiles in nintedanib-treated mice, highlighting the presence of 2 distinct imaging phenotypes (*n*_cluster1_ = 6, *n*_cluster2_ = 4) (Figure 1B). Subanalysis by k-means clustering confirmed their statistical stability (Jaccard coefficients > 0.90) (Supplemental Figure 1, D and E). Intriguingly, these clusters were not discernible through conventional lung densitometry, which showed a significantly (*P* = 0.0157, unpaired Student’s *t* test) reduced tissue density in response to nintedanib treatment, consistent with previous reports (23, 24) (Figure 1, C and D, and Supplemental Figure 1F). Untargeted phosphoproteome quantification in a subset of vehicle- and nintedanib-treated mice 24 hours after the final treatment further confirmed successful and homogeneous target engagement with suppression of key drug-related pathways based on kinase activity enrichment analysis (KAEA), including MTOR and MAP2K1 signaling (25, 26) (Supplemental Figure 1, G and H), thus affirming the efficiency of the drug treatment.

To evaluate if the 2 delta radiomic clusters differ on a molecular level, we performed proteomics analysis. Differential expression analysis of the 7,006 identified proteins in clusters 1 and 2 against the vehicle group uncovered substantial differences between the 2 delta radiomics phenotypes. While 414 proteins (373 downregulated and 41 upregulated) were differentially expressed (DE) in cluster 1, only 169 proteins (127 downregulated and 42 upregulated) showed differential expression in cluster 2 compared with vehicle-treated mice (Supplemental Figure 1, I and J, and Supplemental Tables 1 and 2). Most notably, only minor DE protein overlap (5%) was observed between the 2 clusters (Figure 1, E and F), suggesting that different molecular response phenotypes were captured by delta radiomics.

### Delta radiomic phenotypes reflect differences in molecular response to antifibrotic treatment.

**Figure 2:**
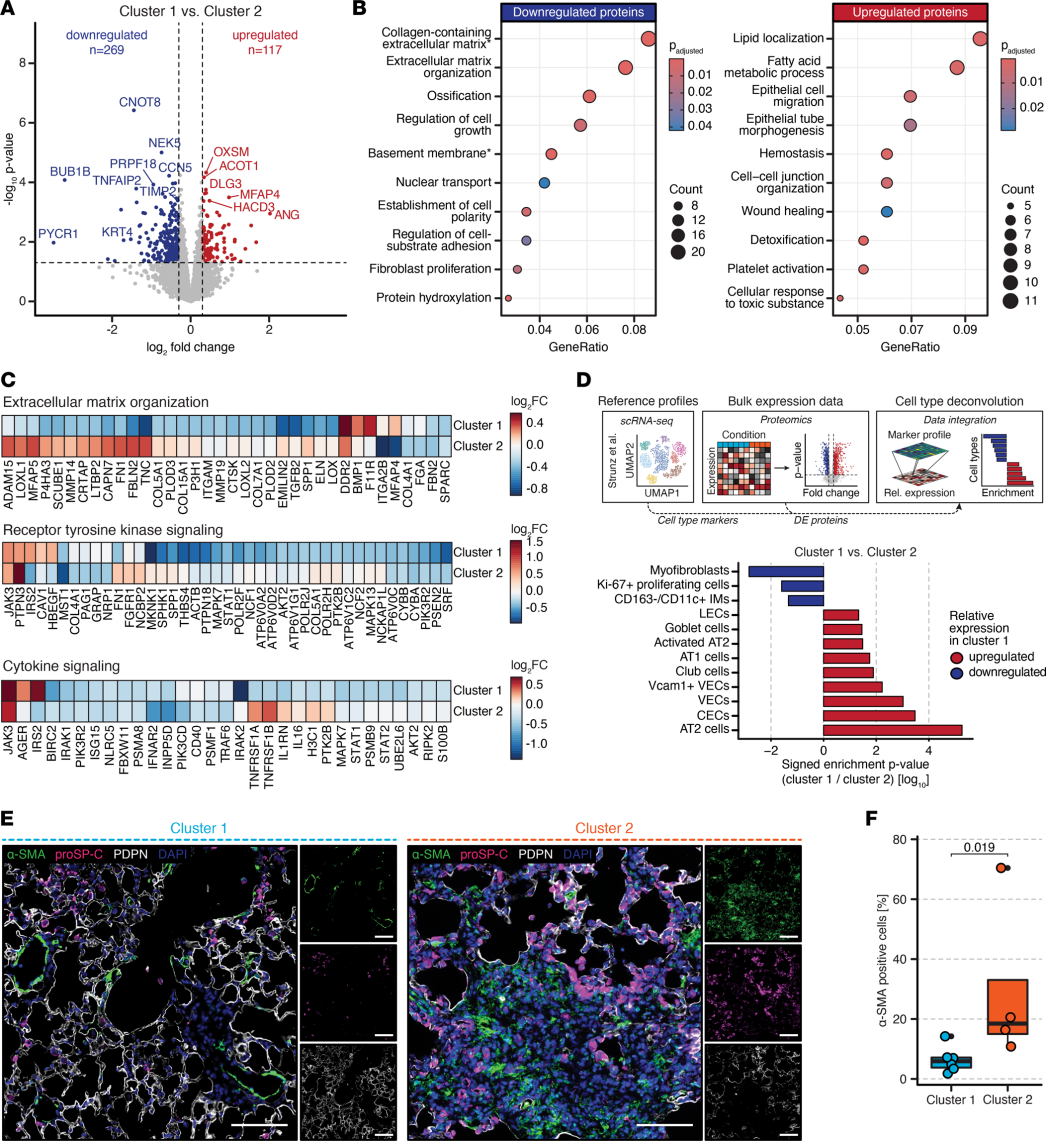
Delta radiomic phenotypes reflect molecular response to antifibrotic treatment. (**A**) Volcano plot of the DE proteins between clusters 1 and 2. Proteins with log_2_ fold change (log_2_FC) > 0.3 and *P* < 0.05 were considered significantly different. Down- and upregulated proteins are highlighted in blue and red, respectively. (**B**) GO pathway analysis of the down- and upregulated DE proteins. Terms marked with an asterisks are of cellular compartment (CC) ontology; all others are of biological process (BP) ontology. (**C**) Heatmap of DE proteins included in Reactome pathways “extracellular matrix organization,” “receptor tyrosine kinase signaling,” and “cytokine signaling” and their expression in clusters 1 and 2 compared with vehicle-treated mice. (**D**) Analysis workflow and bar chart depicting the results from cell type deconvolution analysis. The change of the indicated cell type signature between clusters 1 and 2 is expressed as signed log_10_ enrichment *P* value. (**E**) Representative immunofluorescence stainings of fibrotic regions in clusters 1 and 2. Images show nuclei (DAPI), AT2 cells (proSP-C), myofibroblasts (α-SMA), and AT1 cells (PDPN). Regions are 500 × 500 μm in size. Scale bar: 100 μm. (**F**) Percentage of α-SMA^+^ cells in fibrotic regions of cluster 1 and cluster 2 samples. Mann-Whitney *U* test was used to compare the groups.

To describe the underlying biology of the 2 delta radiomic clusters in closer detail, we analyzed the differences on molecular and cellular level. On the protein level, 386 proteins were differentially regulated between the 2 clusters (Figure 2A and Supplemental Table 3). Gene Ontology (GO) mapping of the downregulated proteins (*n* = 269) revealed enrichment of terms related to profibrotic activity, including extracellular matrix (ECM) organization, regulation of cell growth, and fibroblast proliferation (Figure 2B and Supplemental Table 4). In contrast, the upregulated proteins (*n* = 117) were enriched for pathways related to wound healing and tissue regeneration, including epithelial cell migration and hemostasis (Figure 2B and Supplemental Table 5).

Furthermore, targeted activity analysis of pathways known to be modulated by nintedanib, including ECM organization, receptor tyrosine kinase (RTK) signaling, and cytokine signaling (27), revealed more extensive pathway inhibition in cluster 1 (Figure 2C). Whereas most targets involved in ECM organization (e.g., COL5A1, COL12A1, and TNC) and remodeling (e.g., MMP2, MMP14, TIMP2, and LOX) were downregulated in cluster 1 compared with vehicle, their expression was less changed in cluster 2. Similarly, proteins involved in RTK (e.g., SPP1, STAT1, AKT2, MAPK7, and MAPK13) and cytokine signaling (e.g., IL-6, IRAK1, IRAK2, and PIK3R2) showed higher suppression in cluster 1 than in cluster 2. To independently validate our proteomics results, we performed quantitative PCR (qPCR) of selected gene targets of nintedanib. Aligning with the proteomic observations, we found significant (*P* < 0.05, unpaired Student’s *t* test) suppression of profibrotic (*Col1a1*, *Col3a1*, *Fn1*), proinflammatory (*Il6*, *Spp1*), and nintedanib-targeted (*Tgfb1*, *Cxcl1*, *Tnf*, *Cd40lg*) transcripts in cluster 1 compared with cluster 2 (Supplemental Figure 2).

Preclinical studies demonstrated that nintedanib inhibits myofibroblast differentiation (28), cell proliferation (23), and macrophage activation (29), thereby promoting regeneration of alveolar epithelial cells. To interrogate the cluster-specific effects of nintedanib on the cellular level, we performed cell type deconvolution analysis of our proteomics data as described in ref. 30. This technique quantifies the enrichment of single-cell RNA-Seq–derived cell type marker signatures in bulk cell analysis data such as proteomics or transcriptomics, allowing for the estimation of cell type frequency changes between 2 conditions. Deconvolution revealed lower levels of myofibroblasts, interstitial macrophages, and Ki-67^+^ proliferating cells along with a higher fraction of alveolar type II (AT2) and type I (AT1) lung epithelial cells in cluster 1 compared with cluster 2 (Figure 2D). Tissue immunofluorescence staining for the myofibroblast marker α-smooth muscle actin (α-SMA) together with the AT1 marker podoplanin (PDPN) and the AT2 marker prosurfactant protein C (proSP-C) confirmed a significantly (*P* = 0.019, Mann-Whitney *U* test) lower abundance of α-SMA^+^ myofibroblast infiltrates in fibrotic regions in samples of cluster 1 compared with cluster 2 (Figure 2, E and F).

Taken together, we found that delta radiomics–defined treatment clusters exhibited distinct molecular and cellular characteristics, suggesting a higher degree of response to nintedanib in cluster 1.

### Delta radiomic features reflect changes in disease-relevant molecular pathway activity.

**Figure 3:**
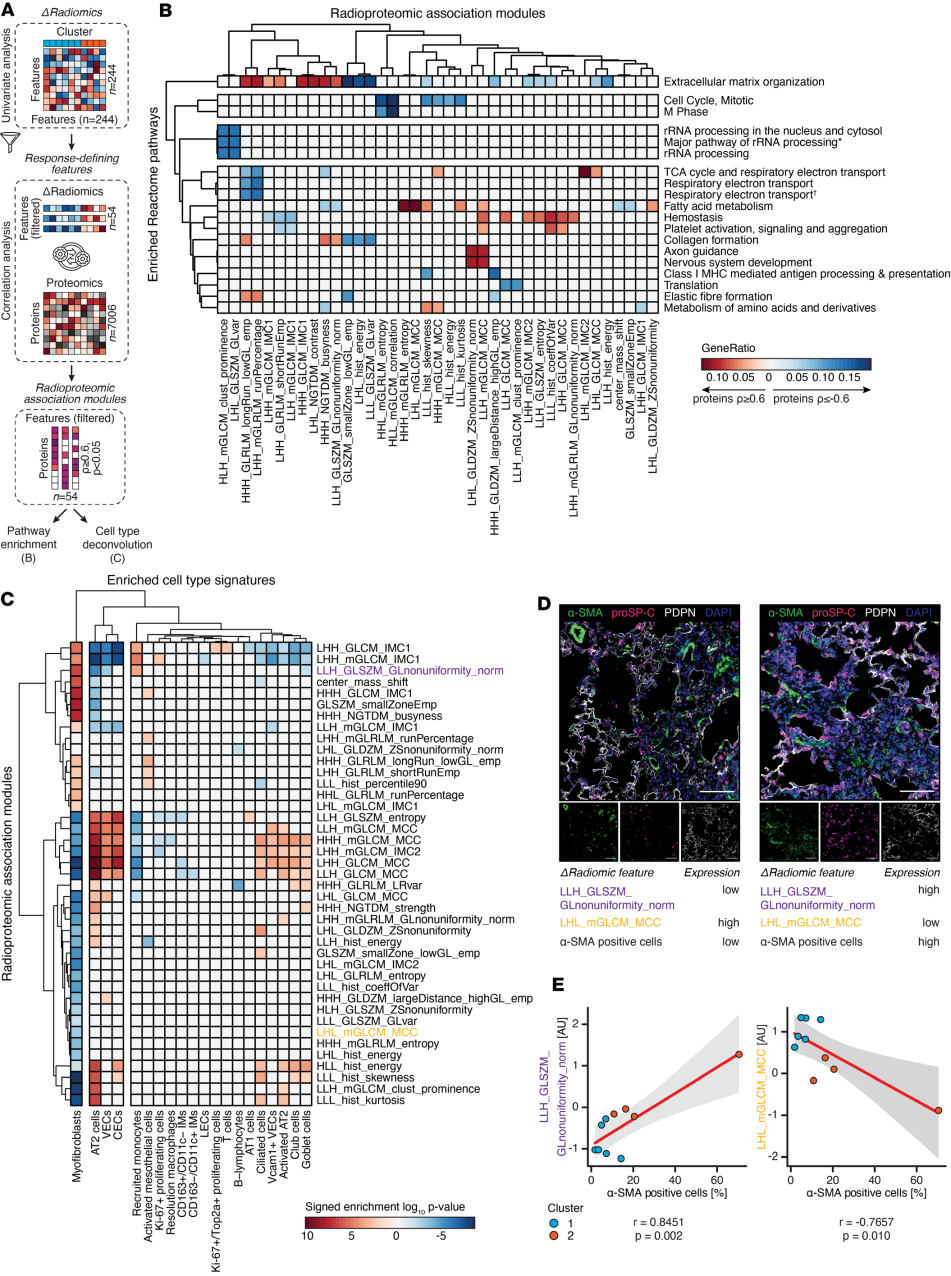
Delta radiomic features reflect changes in disease-relevant molecular pathway activity. (**A**) Workflow schematic. Variable importance of each delta radiomic feature (*n* = 244) for assignment of clusters was assessed by univariate analysis, retaining only “response-defining” features (*n* = 54) with classification score ≥ 0.90. Radioproteomic association modules were compiled by assigning the set of highly correlating proteins (Spearman’s | ρ| ≥ 0.6, P < 0.05) to each response-defining feature. (**B**) Heatmap displaying Reactome pathways enriched (GeneRatio ≥ 0.10, *P*_adj_ < 0.05) in radioproteomic association modules for positively (Spearman’s ρ ≥ 0.6, *P* < 0.05) or negatively (Spearman’s ρ ≤ –0.6, *P* < 0.05) correlating proteins. Only pathways enriched in at least 2 radioproteomic association modules are displayed. Association modules without enriched pathways following filtering are not displayed. Pathway names shortened for R-MMU-6791226 (*) and R-MMU-163200 (†). (**C**) Heatmap displaying cell type signatures enriched (*P* < 0.01) in radioproteomic association modules for positively (Spearman’s ρ ≥ 0.6, *P* < 0.05) or negatively (Spearman’s ρ ≤ –0.6, *P* < 0.05) correlating proteins. Association modules without enriched cell type signatures are not displayed. (**D**) Representative IF stainings of fibrotic lung regions exhibiting a low (left) and high (right) fraction of α-SMA^+^ myofibroblasts. Relative expression of 2 selected delta radiomic features (LLH_GLSZM_GLnonuniformity_norm and LHL_mGLCM_MCC) showing positive or negative enrichment for myofibroblast cell type signatures, respectively, is indicated. Images show nuclei (DAPI), AT2 cells (proSP-C), myofibroblasts (α-SMA), and AT1 cells (PDPN). Scale bar: 100 μm. Each point represents the average fraction of α-SMA^+^ cells of 5 representative fibrotic regions. (**E**) Scatter plot of the Pearson correlation coefficient between the α-SMA^+^ cell fraction quantified by IF and the *Z* scored delta radiomic feature expression of LHL_mGLCM_MCC and LLH_GLSZM_GLnonuniformity_norm. Displayed is the linear model with the best fit (red) together with 95% CIs (gray).

Having established that delta radiomic phenotypes are able to characterize the extent of molecular response to nintedanib treatment, we next investigated the contribution of individual features to noninvasively convey pathway-specific molecular information. To do so, we first identified features promoting cluster separation by analysis of univariate variable importance, resulting in 54 variables with a classification score ≥ 0.90 (Figure 3A and Supplemental Figure 3, A–C). For each of these features, we then established radioproteomic association modules (*n* = 54) by determining the respective correlating protein sets (Spearman’s |ρ| ≥ 0.6, *P* < 0.05) in a sample-matched, cluster-independent approach. Pathway annotation of positively or negatively correlated proteins revealed significant (FDR-adjusted, *P* < 0.05) enrichment of Reactome terms for 45 features, covering 367 unique pathways (Figure 3B and Supplemental Table 6). These findings were replicated through GO database annotation (Supplemental Figure 3D and Supplemental Table 7). Importantly, subsets of association modules were highly distinctive toward specific disease pathophysiology–related pathway activity, including ECM remodeling, cell cycle activity, wound healing, or metabolic processes. K-means clustering of nintedanib-treated samples on features positively correlating with ECM organization (*n* = 8) or hemostasis (*n* = 7) reproduced the original 2 clusters, thereby indicating suppression of ECM remodeling as well as promotion of wound healing in cluster 1 (Supplemental Figure 3E).

To assess if delta radiomic features could provide further insights into changes at the cellular level, we performed cell type deconvolution analysis of the radioproteomic association module–derived protein sets (Spearman’s |ρ| ≥ 0.6, *P* < 0.05) (Figure 3C and Supplemental Table 8). Proteins were ranked by log_10_ *P* value and weighted by correlation coefficient prior to entering deconvolution analysis. Overall, we found 41 response-defining delta radiomic features with significant (*P* < 0.01, Kolmogorov-Smirnov test) cell type marker profile enrichment, accounting for 20 different cell types. Noticeably, myofibroblasts and AT2 cells, as well as vascular and capillary endothelial cell signatures, showed the most significant correlations. Typically, we observed an inverse correlative relationship between profibrotic and proregenerative cell types, as for instance myofibroblasts and AT2 cells. Utilizing immunofluorescence quantification of α-SMA^+^ myofibroblasts, we validated the top positive and negative correlating features, *LLH_GLSZM_GLnonuniformity_norm* (Pearson’s *r* = 0.85, *P* = 0.002) and *LHL_mGLCM_MCC* (Pearson’s *r* = –0.77, *P* = 0.010), which demonstrated significant correlations with myofibroblasts in fibrotic regions (Figure 3, D and E).

Collectively, our results demonstrate that delta radiomic features captured changes of highly specific molecular and cellular information, thereby highlighting their potential as surrogates for molecular treatment response phenotypes.

### Delta radiomics stratifies nintedanib-treated patients with PF-ILD according to lung function decline.

**Figure 4:**
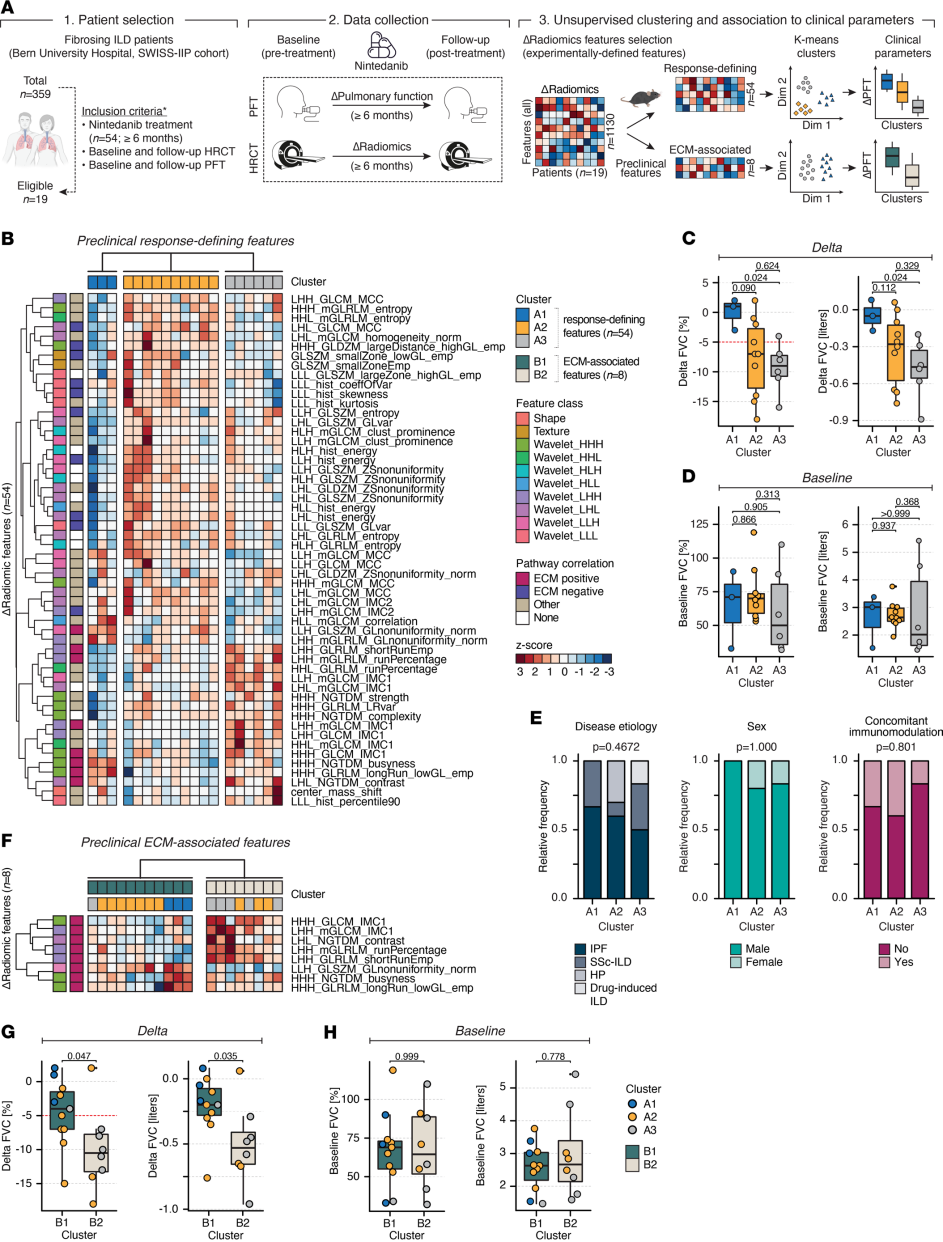
Delta radiomics stratifies nintedanib-treated patients with PF-ILD according to lung function decline. (**A**) Workflow schematic. We retrospectively included patients (*n* = 19 of 359 patients) with PF-ILD undergoing treatment with nintedanib at Bern University Hospital and the SWISS-IIP cohort fulfilling the inclusion criteria. For each patient, changes in pulmonary function parameters and radiomic features were calculated between pre- and posttreatment stages. Unsupervised k-means clustering of patients was performed on subsets of experimentally defined delta radiomic features, including response-defining features (*n* = 54) and features positively enriched for ECM remodeling pathway activity (*n* = 8). The resulting clusters were investigated for differences in clinical outcome parameters and patient demographics. (**B**) Heatmap displaying the results of unsupervised k-means clustering of the *Z*-scored response-defining delta radiomic feature set (*n* = 54) in the PF-ILD cohort. The feature class for each variable and the enrichment of the radioproteomic association module for Reactome pathways is indicated. (**C** and **D**) Box plots comparing FVC (percent predicted and liters) delta and baseline level between clusters A1–A3. Mann-Whitney *U* test was used to compare the groups (**E**) Associations of clusters A1–A3 with clinical and demographic parameters in the PF-ILD cohort. Fisher’s exact test was used to compare the categorical variables. (**F**) Heatmap displaying the results of unsupervised k-means subclustering of the *Z*-scored features whose radioproteomic association modules are positively enriched with ECM remodeling Reactome pathway activity (*n* = 8) in the PF-ILD cohort. The feature class for each variable and the enrichment of the radioproteomic association module for Reactome pathways is indicated. (**G** and **H**) Box plots comparing FVC (% pred and liters) delta and baseline level between subclusters B1 and B2. Mann-Whitney *U* test was used to compare the groups.

We previously demonstrated the high transferability of radiomic signatures from experimental models to human ILD (31). To assess whether our preclinical delta radiomic features could stratify nintedanib-treated patients based on their extent of lung function decline, we retrospectively analyzed delta radiomic feature profiles of 19 patients with PF-ILD who received antifibrotic therapy for a median of 12.2 ± 5.7 months (median ± interquartile range [IQR]) (Figure 4A). ILD etiologies included IPF (*n* = 11), systemic sclerosis–associated ILD (SSc-ILD, *n* = 4), hypersensitivity pneumonitis (HP, *n* = 3), and drug-induced ILD (*n* = 1) (Table 1).

Unsupervised k-means clustering on the preclinical response–defining delta radiomic feature set (*n* = 54) revealed 3 fairly robust (Jaccard indices > 0.60) clusters A1–A3 within the nintedanib-treated PF-ILD cohort (Figure 4B and Supplemental Figure 4A). These clusters stratified patients according to their annual rate of lung functional decline (Figure 4C, Supplemental Figure 4B, and Supplemental Table 9). Within the observational period, cluster A1 showed a significantly (*P* < 0.05, Mann-Whitney *U* test) lower forced vital capacity (FVC) decline (1.0% ± 2.5%, –50 ± 125 mL; median ± IQR) compared with cluster A3, which showed a substantial decline (–9.0% ± 3.5%, –465 ± 225 mL, median ± IQR). Cluster A2 presented with an intermediate phenotype with a considerable FVC decline (–7.0% ± 10.0%, –280 ± 450 mL, median ± IQR), although it was not statistically different from clusters A1 and A3 (*P* > 0.05, Mann-Whitney *U* test). Notably, no significant differences (*P* > 0.05, Mann-Whitney *U* or Fisher’s Exact test) between the clusters were observed for pretreatment FVC levels (Figure 4D), disease etiology, sex, smoking status, disease duration, presence of concomitant immunomodulatory therapy, or presence of pulmonary (arterial) hypertension (Figure 4E and Supplemental Figure 4C).

Our preclinical results reveal subsets of radioproteomic association modules that were specifically linked to ECM remodeling activity, the key molecular target of antifibrotic therapy (27). To evaluate if the corresponding delta radiomic features (*n* = 8, positive enrichment) would lead to improved stratification, we performed k-means clustering of the PF-ILD cohort using this feature subset (Figure 4F and Supplemental Figure 4D). The resulting 2 stable clusters B1 and B2 (Jaccard indices > 0.75) exhibited significant differences (*P* < 0.05, Mann-Whitney *U* test) on delta FVC level, with cluster B1 (–4.0% ± 5.5%, –200 ± 205 mL, median ± IQR) displaying less functional decline compared with cluster B2 (–10.5% ± 5.5%, –530 ± 245 mL, median ± IQR). Notably, this effectively redefined the previous intermediate cluster A2 into either cluster B1 or B2, corresponding to low/intermediate and high FVC decline (Figure 4G and Supplemental Table 10). Similar to our previous findings, we found no significant differences between clusters B1 and B2 for pretreatment FVC levels (*P* > 0.05, Mann-Whitney *U* test) (Figure 4H) or demographic and clinical variables (*P* > 0.05, Fisher’s exact test) (Supplemental Figure 4, E and F).

## Discussion

Accurate monitoring of response to antifibrotic therapy is an urgent need for effective management of patients with PF-ILD. However, differentiation between natural disease progression and treatment failure is difficult by means of conventional pulmonary function test (PFT) and HRCT assessment (7). Molecular response markers, including peripheral blood biomarkers, may improve precision, but these are still in early developmental stages and may not fully reflect lung tissue phenotypes (8, 32, 33). Over the last decade, radiomics has emerged as a powerful tool for drug response monitoring and predicting outcomes in various diseases, such as cancer, neurological disorders, and, recently, also ILDs (34–36). The strength of radiomics lies in its ability to provide integrated information on whole lung tissue pathology, conveying both structural and molecular information (16, 18, 37).

In this study, we employed an integrative radioproteomics approach to demonstrate that CT-based delta radiomic profiling can noninvasively stratify the molecular response to nintedanib treatment in the preclinical bleomycin-induced lung fibrosis model. Our analysis identified 2 molecular response clusters based on delta radiomic profiles, which were not distinguishable through conventional histogram-based CT measure. We discovered distinct radioproteomic association modules that conveyed disease- and drug-specific biological pathway activities and cell type signatures, including ECM remodeling, hemostasis, and fibroblast activation. Evaluation of the preclinical response–defining delta radiomic features — in particular, the ECM-associated features — in a nintedanib-treated PF-ILD cohort accurately stratified patients according to their extent of lung function decline.

Previous reports have shown the potential of CT-derived imaging characteristics for assessing the response to antifibrotic treatment. Lung attenuation histogram–derived measures, for example, have proven reliable in studying the efficacy of antifibrotic drugs in preclinical lung fibrosis models (24, 38, 39). However, their use as surrogate markers is mostly limited to macroscopic tissue pathologic properties, falling short of resolving the underlying molecular landscape, as also evidenced in the current study. In addition, these variables represent the summary of gray level intensities not taking the spatial interrelationship of voxels into account. This potentially limits their sensitivity to capture the subtle changes induced by antifibrotic treatment in patients with PF-ILD who often present with morphologically complex and heterogeneous disease patterns (40, 41). In contrast, higher-order radiomic variables, such as texture features, quantify the spatial variations in image characteristics, offering added information for treatment monitoring. Utilizing a texture-based quantitative lung fibrosis (QLF) score, Kim et al. were able to stratify patients with IPF undergoing experimental antifibrotic treatment according to the rate of pulmonary function decline (42). Furthermore, Devkota et al. showed that texture-derived nanoradiomics, and not conventional quantitative CT features, captured treatment-induced changes of cellular therapy in tumor xenografts (17).

The added and complementary value of radiomics arises from the integrated in-depth analysis of tissue heterogeneity across spatial scales, thereby conveying pathophysiological information of the whole organ. Imaging omics approaches, including radiogenomics, radiotranscriptomics, and radioproteomics, investigate the association between macroscopic radiomic and microscopic molecular features derived from genomic, transcriptomic, or proteomic profiling, respectively, to define the underlying biological basis of imaging phenotypes and derive noninvasive imaging surrogates for molecular profiles (43). So far, imaging omics have nearly exclusively been studied in the context of cancer. For instance, recent studies utilized radiogenomics to unravel intratumoral heterogeneity phenotypes in multicenter breast cancer cohorts (19, 44) and identified activated ferroptosis pathways to be associated with high tumor heterogeneity (19). Moreover, radiogenomics has been employed to noninvasively characterize the biological activities of specific breast cancer subclones (44).

In this study, we provide evidence that delta radiomic signatures are sensitive toward antifibrotic therapy–induced molecular changes in experimental fibrosing ILD. We add novelty by integrating delta radiomics with proteomics and utilizing the resulting association modules to functionally explain different treatment response phenotypes on a pathophysiologic level. The ability to assess distinct pathway and cellular activities noninvasively from standard-of-care HRCT scans could pave the way toward digital molecular disease fingerprints that could inform precision medicine (45).

Our study has some limitations. Firstly, in our preclinical studies, the absence of pretreatment proteome profiles in mice did not allow us to investigate the molecular landscape at therapy start, which may have confounded the antifibrotic treatment response. However, unlike human disease, interindividual variance of lung fibrosis development in mice is considered to be low in presence of high bleomycin doses (46–48). Future validation of our findings in independent lung fibrosis models will be necessary to ensure the broader applicability of our approach, which currently is limited to using only 1 mouse model with uniform delivery of bleomycin and treatment compound. Secondly, using only female mice may limit its ability to account for potential sex-related differences in treatment response. Thirdly, generalizability of our findings to human ILD is limited by the pilot character and retrospective nature of our study with bias toward IPF as most frequent PF-ILD etiology. Although we could not find statistically significant differences between etiologies and in potential confounders, we cannot rule out that those factors, particularly concomitant immunomodulatory therapy, may have contributed to the effects observed on a delta radiomic level given the relatively small sample size. Furthermore, the lack of pre- and posttreatment biosamples precluded molecular validations. Future prospective multicenter studies that include the collection of liquid biopsies for molecular evaluation, together with the inclusion of a placebo group, will be necessary to fully elucidate the applicability of delta radiomic signatures as a digital fingerprint for disease- or drug-response monitoring. Nonetheless, our ability to detect significant changes in the extent of pulmonary function decline based on preclinical functionally described delta radiomic features in this small but well-defined cohort showcases the method’s inherent potential. Finally, due to the small sample sizes, we could not yet assess the predictive potential of baseline radiomic profiles for treatment response, which will be the subject of future studies.

In conclusion, this study highlights delta radiomics as a noninvasive tool to stratify response to antifibrotic treatment in experimental fibrosing ILD through its ability to decode tissue-underlying molecular information. Its potential for transferability to human disease is a first step toward precision medicine, facilitating individual therapy monitoring and risk-benefit assessment in the context of lifelong therapies.

## Methods

Supplemental Methods are available online with this article.

### Sex as biological variable.

The patients in this study were retrospectively selected irrespective of biological sex and included both females and males with PF-ILD. Our study examined female mice in the bleomycin-induced lung fibrosis model (a) to reproduce the setup of the published preclinical in vivo nintedanib trials (28) and (b) because of animal well-being considerations, where male mice are known to exhibit higher disease severity compared with female mice and, thus, a higher mortality rate (49).

### Animal experimentation and ethics statement.

Lung-derived delta radiomic profiles were studied upon treatment with nintedanib in the well-established murine bleomycin-induced fibrosing ILD model (23, 28). Briefly, lung fibrosis was induced in C57BL/6J mice (*n* = 30, female, 8 weeks old) by intratracheal (i.t.) instillation with 2 U/kg bleomycin sulfate (18). Mice were randomized into study groups and treatment with 60 mg/kg nintedanib (*n* = 15), or vehicle-only (deionized water, *n* = 15) was provided once daily per os (p.o.) from days 7–20 in a double-blinded manner. Lung μCT scans (SkyScan 1176) of each animal were acquired before (day 7) and after treatment (day 21) as previously described (18). All mice were sacrificed 24 hours after the final treatment, followed by exsanguination, transcardial perfusion, and collection of the lung tissue for molecular analysis. Mice were excluded from further analysis if humane endpoints were reached (*n* = 3) or if severe lung abnormalities, including atelectasis or unilateral fibrosis development, were evident on μCT scans (*n* = 3). The final sample size for nintedanib- and vehicle-treated mice was *n* = 10 and *n* = 14, respectively.

### Patient cohort, clinical data, and ethics statement.

We validated our experimental findings in a retrospectively selected PF-ILD cohort of 19 patients from the Bern University Hospital and the SWISS-IIP cohort that were undergoing treatment with nintedanib. In total, 359 patients were screened for the following eligibility criteria: (a) diagnosis of PF-ILD (50), including IPF, SSc-ILD, HP, or drug-induced ILD; (b) treatment with nintedanib (≥ 100 mg twice daily; ≥ 6 months); (c) availability of pre- and posttreatment HRCT scans fulfilling the predefined quality criteria (Supplemental Methods); (d) pre- and post-treatment PFT recording fulfilling the predefined quality criteria (supplementary material); and (e) absence of secondary lung diseases at times of HRCT and PFT recordings. In total, 54 of 359 patients received nintedanib treatment for ≥ 6 months, with 19 fulfilling also the remaining inclusion criteria. Summaries of patient demographics and clinical characteristics, and the HRCT scan acquisition parameters, are provided in Table 1 and Supplemental Table 11.

### Delta radiomics calculation.

Calculation of radiomic features was performed on semiautomatically segmented lungs using Z-Rad software (v.7.3.1, https://medical-physics-usz.github.io/) as previously described (18). Mouse lungs were resized to isotropic voxels of 0.15 mm. To achieve a comparable voxel size in patients, human lungs were resized to isotropic voxels of 2.75 mm, corresponding to an estimated 6,000-fold volumetric difference (51). Both mouse and human lung volumes were discretized to a fixed bin size of 50 Hounsfield units (HU) in a range of –1,000 HU to 200 HU. From the resized volumes, 1,388 radiomic features were calculated per lung scan and time point, corresponding to histogram (*n* = 17), texture (*n* = 137), shape (*n* = 2), and wavelet-transformed features (*n* = 1,232). Delta radiomic features describing the change of each feature between pre-and posttreatment were expressed as Δ values: ΔFeature = Feature (t_2_) – Feature (t_1_) (52). Lung density measurement was inferred from the radiomic lung attenuation histogram-derived feature *hist_mean*.

### Proteomics and phosphoproteomics.

For proteomics and phosphoproteomics, the middle lobe of the right mouse lung was snap frozen in liquid nitrogen and stored at –80°C until processing. Sample preparation and mass spectrometry profiling was performed at the PMSCF at the University of Bern using standard protocols. For comparative proteomics, all vehicle- (*n* = 14) and nintedanib-treated (*n* = 10) samples were analyzed. One vehicle sample was excluded from analysis due to issues in sample preparation. Differential expression of proteins between groups of interest was calculated in R using the “limma” package with standard settings. For phosphoproteomics, randomly selected vehicle- (*n* = 5) and nintedanib-treated (*n* = 5) sample subsets were analyzed. Differential expression of phosphosites and subsequent KAEA was performed as described in ref. 53.

### Gene expression analysis.

Total RNA was isolated from blood-free cranial lobes of the right mouse lung using the RNeasy Tissue Mini Kit (Qiagen). Isolated RNA was reverse transcribed into cDNA using the Transcriptor First Strand cDNA Synthesis Kit (Roche Diagnostics). Expression of selected nintedanib target genes was analyzed by SYBR Green qPCR as described in ref. 54. Expression of mRNA was expressed as ΔCt values with *Rplp0* as reference gene. Fold changes were calculated using the ΔΔCt method. A list of the primer pairs is provided in Supplemental Table 12.

### Immunofluorescence and microscopy.

Formalin-fixed paraffin-embedded lung sections of 3 μm thickness were deparaffinized, followed by heat-mediated antigen retrieval and blocking for nonspecific antibody binding with 5% BSA. Incubation with primary antibodies was performed overnight at 4°C, followed by incubation with secondary fluorescence-labeled antibodies for 2 hours at room temperature. Nuclei were visualized by counterstaining with DAPI for 10 minutes at room temperature. Antibodies and dilutions are listed in Supplemental Table 13. Microscopic imaging was performed with an AxioScan.Z1 slide scanner (Zeiss) using a Plan-Apochromat 20×/0.8 M27 objective. Cells positively stained for α-SMA were quantified using the “Positive cell detection” tool of the open source software QuPath (v.0.4.0). From each sample, 5 representative areas at 500 × 500 μm were analyzed and the sample average was used for statistical analyses.

### Unsupervised clustering.

Unsupervised agglomerative hierarchical or k-means clustering of *Z* scored features was performed to identify subgroups of mice or patients with similar delta radiomic feature patterns. Clusterability was evaluated by Hopkin’s statistic H. The optimal number of clusters was determined by average silhouette statistic. Stability of the resulting clusters was assessed by Jaccard bootstrapping.

### Variable importance evaluation.

The importance of each delta radiomic feature cluster assignment by unsupervised clustering was calculated by filter-based variable importance, retaining only features with classification score ≥ 0.9 (Supplemental Table 14).

### Radioproteomic correlation analysis.

Spearman’s rank correlation coefficient ρ was calculated between delta radiomic feature subsets and the log_2_-transformed expression intensity of every protein, retaining only proteins with *P* < 0.05 and ρ ≥ 0.6 to establish radioproteomic association modules. Pearson’s correlation coefficient *r* was calculated between delta radiomic feature subsets and the fraction of α-SMA^+^ cells.

### GO and Reactome pathway enrichment.

Lists of DE proteins or radiomics-correlated proteins were entered into GO or Reactome pathway enrichment analysis, retaining results after FDR adjustment (*P* < 0.05).

### Cell type signature enrichment analysis.

To infer relative cell type frequency changes between 2 groups from proteomics data, we applied signature enrichment analysis (30, 55), utilizing their single-cell marker gene data set. Cell type signatures were defined as sets of genes with cell type–specific gene expression of log_2_ fold change > 0.3 and adjusted *P* < 0.05.

### Statistics.

All statistical analyses were performed and visualized in R (v.4.3.1). The specific statistical analyses used are described in the figure legends and include unpaired, 2-tailed Student’s *t* tests, 2-tailed Mann-Whitney *U* tests, 2-tailed Kolmogorov-Smirnov tests, and Fisher’s exact tests. For all analyses, *P* < 0.05 was considered statistically significant unless stated otherwise.

### Study approval.

Ethical approval for animal experimentation was granted by the Swiss cantonal veterinary office (license no. ZH082/2021), and experimentation was performed in strict compliance with Swiss animal protection laws and guidelines. Ethical approval for use of human patient data was granted by the local Swiss ethics committees (BASEC-ID: 2023-01920 [ILDALMO]; PB_2016_01524 [SWISS-IIP]). Informed written consent was obtained from all patients.

### Data availability.

Data and code for reproduction of the main findings of this study are available at Zenodo (https://doi.org/10.5281/zenodo.11395642). Individual data values associated with this study are reported in the Supporting Data Values file. The mass spectrometry proteomics data have been deposited to the ProteomeXchange Consortium via the PRIDE (56) partner repository with the data set identifier PXD052594.

## Author contributions

DL and JGS conceived and designed the study, acquired, analyzed and interpreted the data, designed the figures, and wrote the manuscript. CYM, LRK, GMC, ACS, MFC, and LE performed the acquisition and analysis of patient data. HW performed immunofluorescence stainings. MB contributed to the acquisition and analysis of animal and patient data. MC, HSG, STL, KK, OD, and SEV provided intellectual input. ACU and MH contributed to the acquisition and analysis of proteome data. BM contributed to the design and conception of the study and wrote the manuscript. All authors read the final manuscript.

## Supplementary Material

Supplemental data

Supplemental tables 1-8

Supporting data values

## Figures and Tables

**Table 1:**
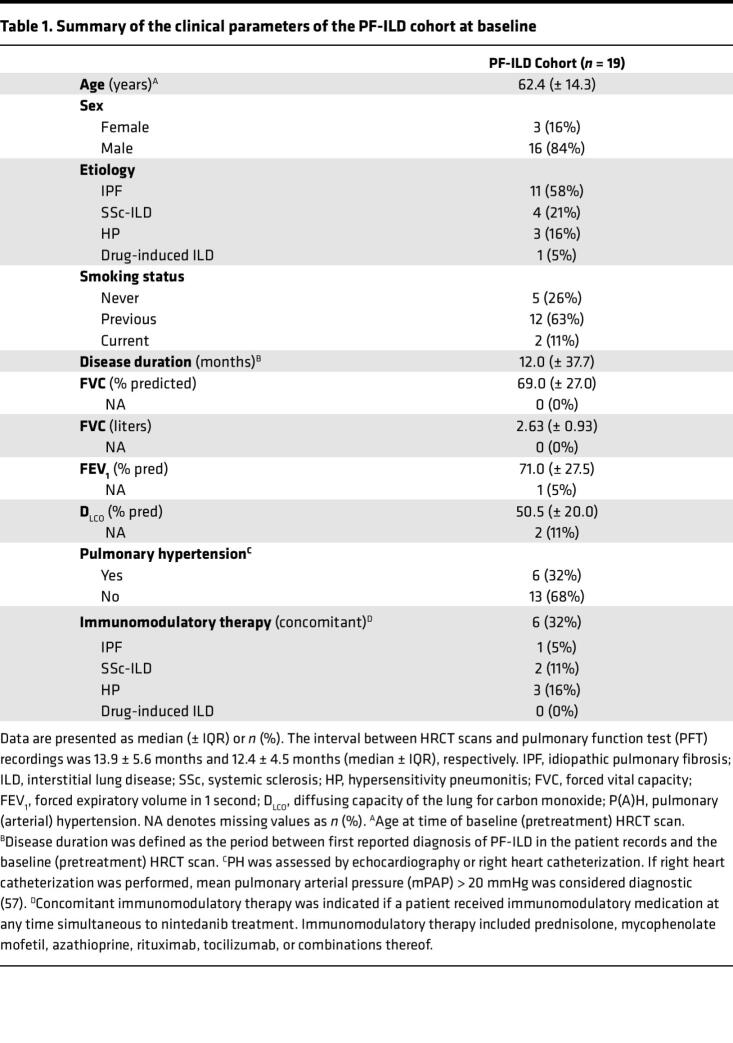
Summary of the clinical parameters of the PF-ILD cohort at baseline

## Version 1 07/16/2024

In-Press Preview

## Version 2 08/08/2024

Electronic publication
