# Supplementary material for: Radioproteomics stratifies molecular response to antifibrotic treatment in pulmonary fibrosis
Source: JCI Insight. 2024 Jul 16;9(15):e181757. doi: 10.1172/jci.insight.181757 (PMC11383602; doi:10.1172/jci.insight.181757)
Supplement: Supplemental data [file jciinsight-9-181757-s264.pdf]

Supplementary Material

Radioproteomics stratifies molecular response to antifibrotic treatment in pulmonary fibrosis

David Lauer[1,2,3], Cheryl Y. Magnin[1,2], Luca Kolly[1,2], Huijuan Wang[1,2], Matthias Brunner[1,2], Mamta Charbria[4], Grazia M. Cereghetti[5], Hubert S. Gabryś[6], Stephanie Tanadini-Lang[6], Anne-Christine Uldry[7], Manfred Heller[7], Stijn E. Verleden[8], Kerstin Klein[1,2], Adela-Cristina Sarbu[1], Manuela Funke-Chambour[2,9], Lukas Ebner[5,10,11], Oliver Distler[3], Britta Maurer[1,2], Janine Gote-Schniering[1,2,9]

[1] Department of Rheumatology and Immunology, Inselspital, Bern University Hospital, University of Bern, Bern, Switzerland. [2] Lung Precision Medicine (LPM), Department for BioMedical Research (DBMR), University of Bern, Bern, Switzerland. [3] Department of Rheumatology, Center of Experimental Rheumatology, University Hospital Zurich, University of Zurich, Zurich, Switzerland. [4] Department of Health Sciences and Technology, ETH Zurich, Zurich, Switzerland. [5] Department of Diagnostic, Interventional, and Pediatric Radiology, Inselspital, Bern University Hospital, University of Bern, Bern, Switzerland. [6] Department of Radiation Oncology, University Hospital Zurich, Zurich, Switzerland. [7] Proteomics & Mass Spectrometry Core Facility, Department for BioMedical Research (DBMR), University of Bern, Bern, Switzerland. [8] Department of ASTARC, University of Antwerp, Antwerp, Wilrijk, Belgium. [9] Department of Pulmonary Medicine, Allergology and Clinical Immunology, Inselspital, Bern University Hospital, University of Bern, Bern, Switzerland. [10] Department of Radiology, Cantonal Hospital Lucerne, Luzern, Switzerland. [11] Institute for Radiology, Hirslanden Bern Klinik Beau-Site, Bern, Switzerland.

Correspondence: Janine Gote-Schniering, PhD
Lung Precision Medicine, Department for BioMedical Research, University of Bern, 3008 Bern, Murtenstrasse 28, Switzerland. Mail: janine.gote-schniering@unibe.ch. Phone: +41 31 684 04 06.

Supplementary Figures

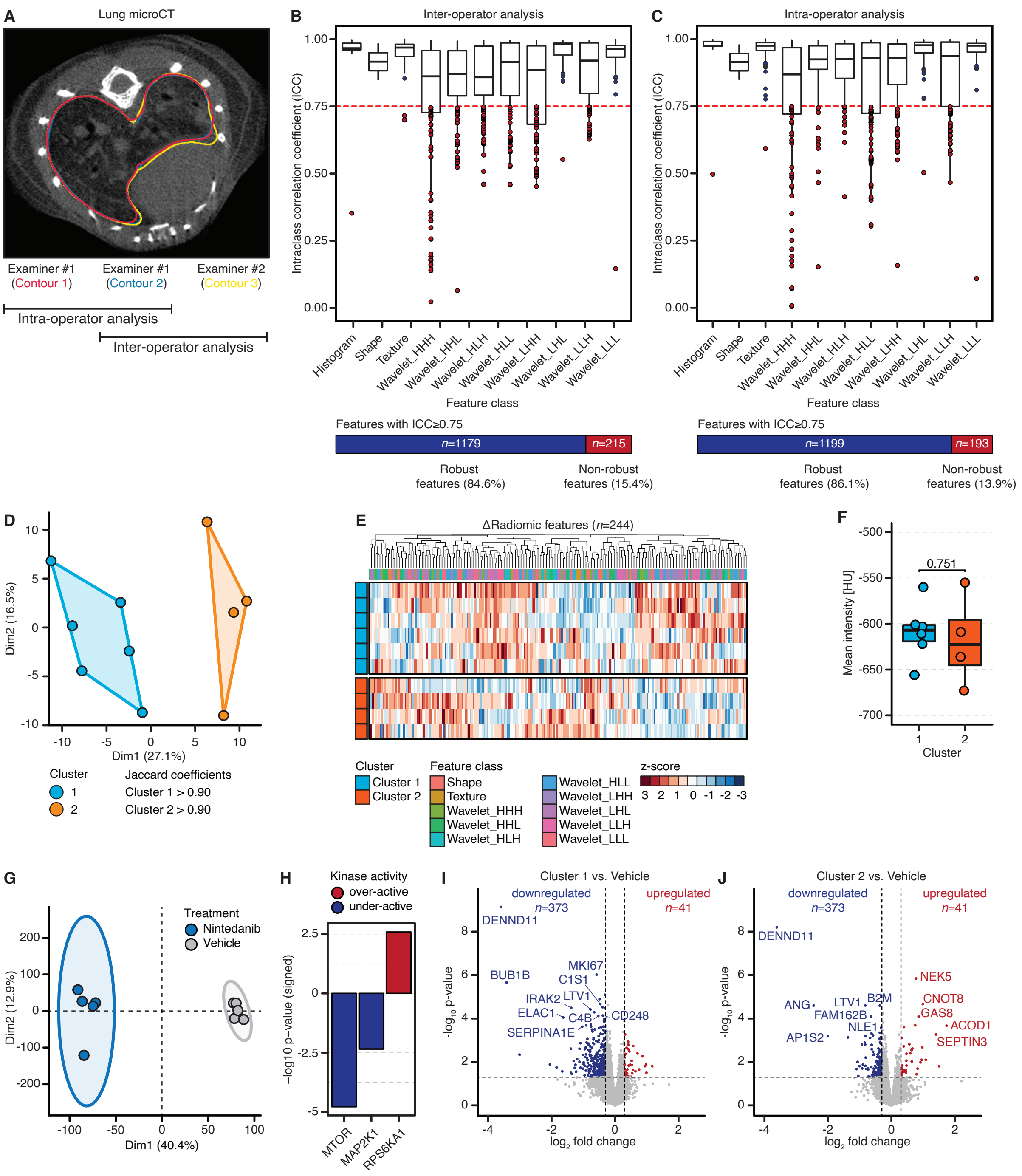

Supplementary Figure 1. (**A**) Evaluation of the radiomic feature stability against inter- and intra-reader variation in the semi-automated lung segmentation workflow. Displayed is a representative transversal microCT image of a bleomycin-induced mouse lung. Outlined are three semi-automatically delineated lung contours of two different examiners (examiner 1: red and blue; examiner 2: yellow). For intra- and inter-operator intraclass correlation coefficient (ICC) analysis, a total of *n*=16 randomly selected lung scans covering different time points were segmented in this manner. (**B**) Boxplots displaying the distribution of the ICC coefficient per radiomic feature class for inter-operator ICC analysis and (**C**) intra-operator ICC analysis. The red dashed line indicates the set ICC threshold at 0.75. The stacked bar charts summarize the relative frequency and total number of robust and non-robust radiomic features.

(**D**) K-means clustering of z-scored delta radiomic features (*n*=244) of nintedanib-treated mice (*n*=10) shows two stable clusters (Jaccard coefficients>0.90, where 1 describes perfect stability). (**E**) Heatmap summary of the k-means clustering results (nintedanib-treated mice, *n*=10). Clusters and the feature class of each variable are indicated. (**F**) Lung tissue density in cluster 1 and 2 expressed as mean Hounsfield unit (HU) intensity post-treatment. Mann-Whitney U-test was used to compare groups. (**G**) Principal component analysis of the phosphosite expression values (*n*=20043) in subsets of randomly selected nintedanib- (*n*=5) and vehicle-treated (*n*=5) mice. (**H**) Kinase activity enrichment analysis (KAEA) of differentially expressed phosphosites in nintedanib- against vehicle-treated mice. Under-active kinases are colored in red, over-active kinases are colored in blue. (**I**) Volcano plots of protein expression in cluster 1 and (**J**) cluster 2 compared to vehicle-treated mice. Proteins with $\log_2$FC>0.30 and p<0.05 were considered to be differentially expressed. Down- and upregulated proteins are highlighted in blue and red, respectively.

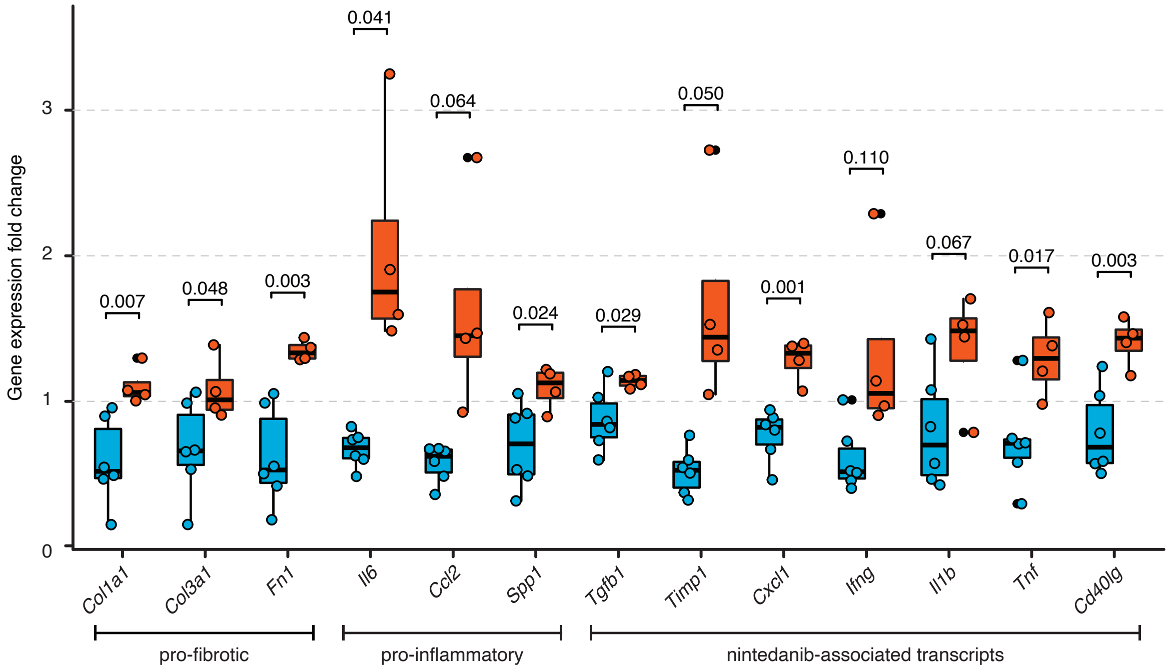

Supplementary Figure 2. Quantitative PCR of pro-fibrotic (*Col1a1*, *Col3a1*, *Fn1*), pro-inflammatory (*Il6*, *Ccl2*, *Spp1*), and nintedanib-targeted (*Tgfb1*, *Timp1*, *Cxcl1*, *Ifng*, *Il1b*, *Tnf*, *Cd40lg*) genes. Displayed is the mRNA fold change expression using the delta-delta Ct method ($2^{-\Delta\Delta Ct}$) in cluster 1 (blue) and cluster 2 (red) compared to vehicle-treated samples. Each data point represents the mean of two technical replicates. Unpaired Student's t-test was used to compare the groups.

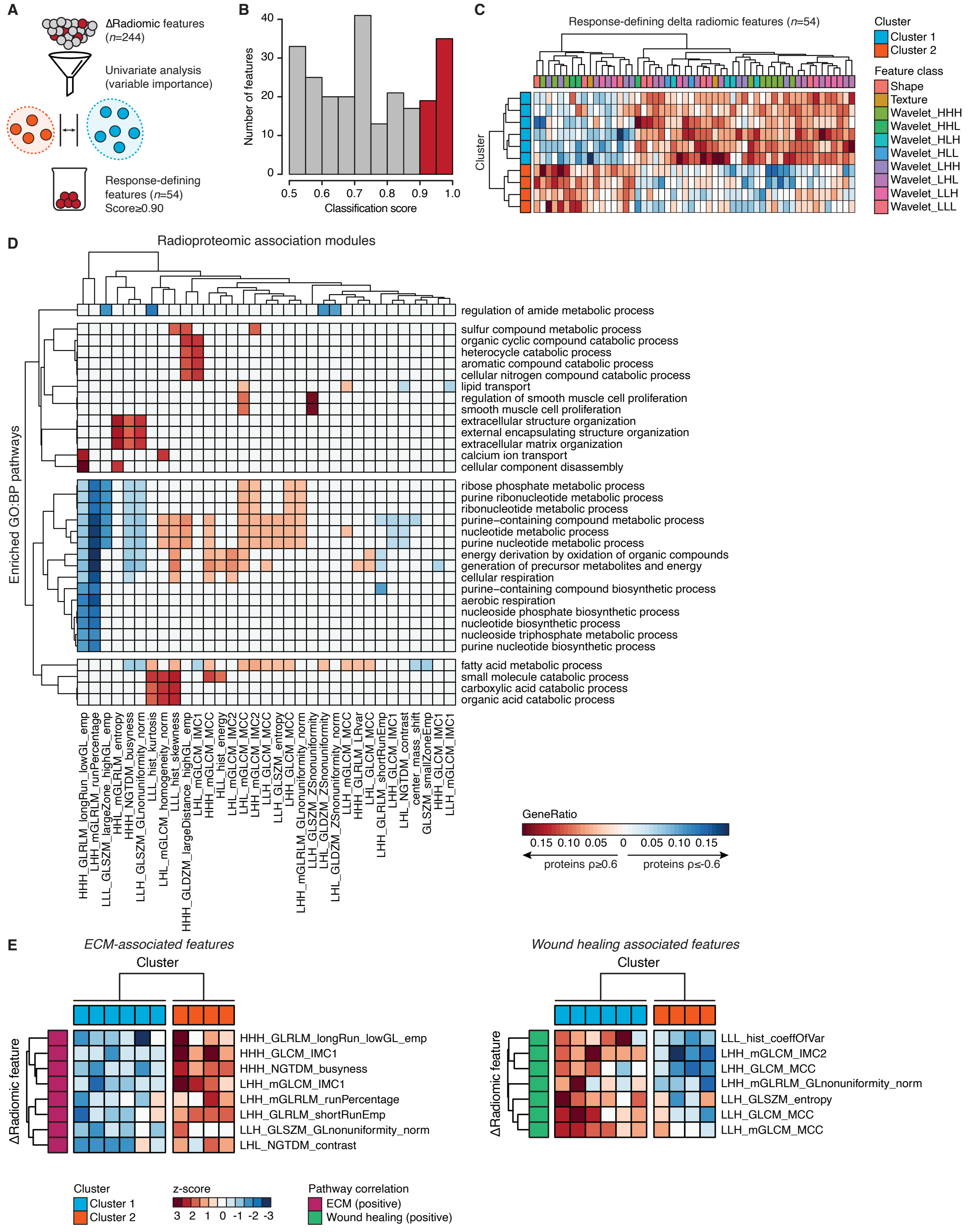

Supplementary Figure 3. Identification of response-defining delta radiomic features and their correlation with disease-relevant pathways. (**A**) Variable importance of each delta radiomic feature (*n*=244) for assignment of clusters was assessed by univariate analysis, retaining only "response-defining" features (*n*=54) with classification score≥0.90. (**B**) Histogram of the univariate analysis results of the delta radiomic features (*n*=244) for classification of cluster assignment. Variables with classification score≥0.90 (red) were considered to have response-defining properties. (**C**) Heatmap displaying the results of the unsupervised hierarchical clustering of the z-scored subset of response-defining delta radiomic features (*n*=54) in nintedanib-treated mice (*n*=10). Cluster assignment and the

feature class of each variable are indicated. (**D**) Heatmap displaying Gene Ontology Biological Process (GO:BP) pathways enriched (GeneRatio≥0.10, adjusted $p<0.05$) in radioproteomic association modules for positively (Spearman's $\rho \geq 0.6$, $p<0.05$, red annotation) or negatively (Spearman's $\rho \leq -0.6$, $p<0.05$, blue annotation) correlating proteins. Only pathways enriched in at least two radioproteomic association modules are displayed. Association modules without enriched pathways following filtering are not displayed. (**E**) Heatmaps displaying the results of unsupervised k-means clustering of z-scored subsets of delta radiomic features positively enriched in extracellular matrix (ECM) remodeling (*n*=8) and wound healing (*n*=7) in nintedanib-treated mice (*n*=10), respectively. Cluster assignment of samples and Reactome pathway enrichment of variables are indicated.

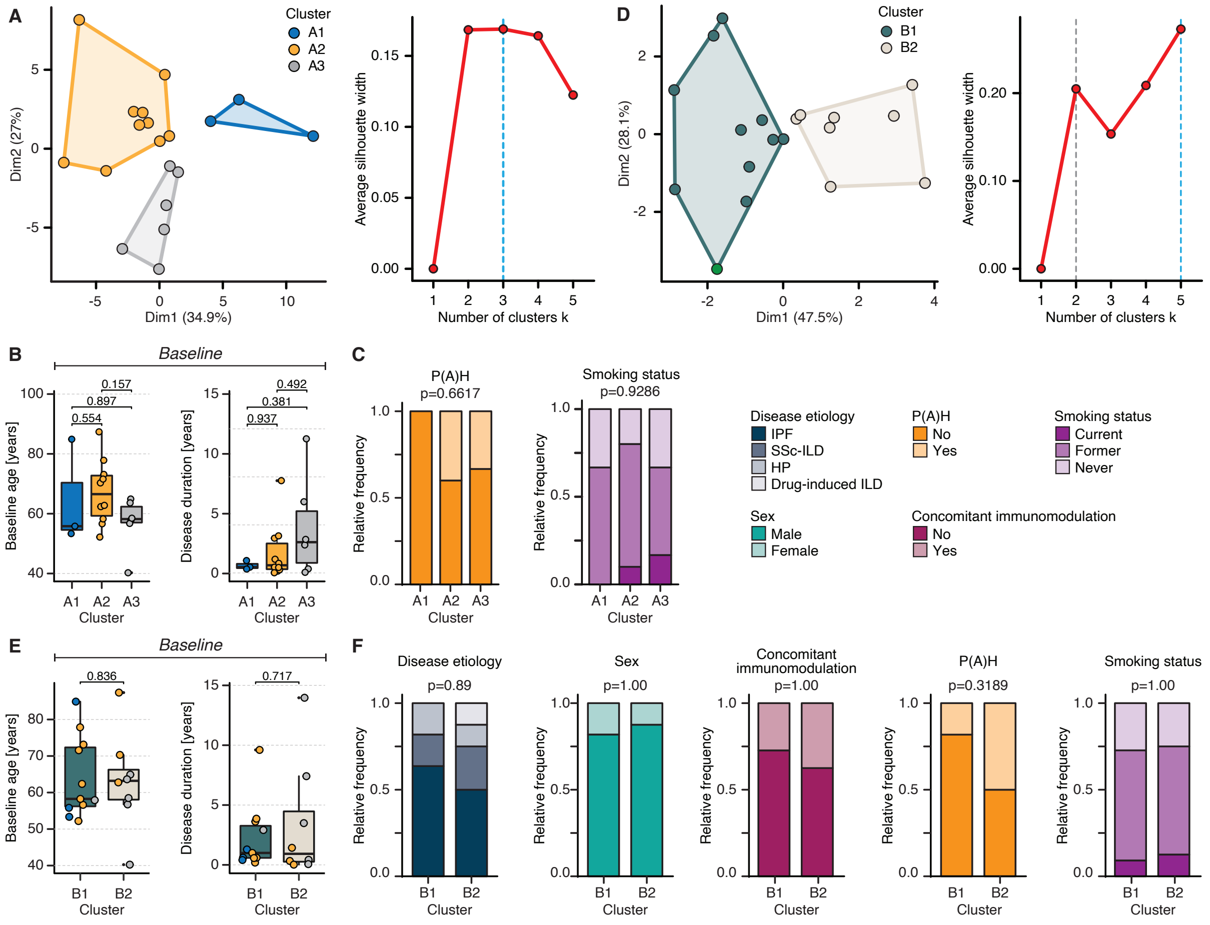

Supplementary Figure 4. Delta radiomics stratifies the degree of pulmonary function decline in nintedanib-treated PF-ILD patients. (**A**) Left: K-means cluster plot for z-scored preclinical response-defining delta radiomic features (*n*=54) of the PF-ILD cohort (*n*=19) indicates three fairly robust clusters (Jaccard coefficients>0.60, where 1 describes perfect stability). Right: Scatter plot showing the average silhouette coefficient versus the number of clusters for the k-means clustering input data. The blue dashed line indicates the global optimum. (**B**) Box plots comparing age (years)# and disease duration (years)¶ at baseline between clusters A1-A3. Mann-Whitney U test was used to compare the groups. (**C**) Associations of clusters A1-A3 with clinical and demographic parameters in the PF-ILD cohort. Fisher's exact test was used to compare the categorical variables. (**D**) Left: K-means cluster plot for z-scored delta radiomic features positively correlating with ECM-remodeling (*n*=8) of the PF-ILD cohort (n=19) indicates two stable clusters (Jaccard coefficients>0.75, where 1 describes perfect stability). Right: Scatter plot showing the average silhouette coefficient versus the number of clusters for the k-means clustering input data. The blue and red dashed line indicate the global and local optimum, respectively. (**E**) Box plots comparing age (years)# and disease duration (years)¶ at baseline between clusters B1 and B2. Mann-Whitney U test was used to compare the groups. #Age is defined as the period between birth date and baseline HRCT scan. ¶Disease duration is defined as the period between the first reported diagnosis of PF-ILD and baseline HRCT scan. (**F**) Associations of clusters B1 and B2 with clinical and demographic parameters in the PF-ILD cohort. Fisher's exact test was used to compare the categorical variables.

Supplementary Tables

Supplementary Table 1. Differentially expressed proteins in nintedanib-treated mice in cluster 1 compared to vehicle-treated mice. The list includes all identified 7006 proteins. Only proteins with $|log_2FC|>0.3$ and $p<0.05$ were considered for analysis. Each entry is described by log_2FC, confidence intervals, average expression, t-value, p-value, B-value, Entrez ID, and gene symbol. Data provided in supplemental tables file.

Supplementary Table 2. Differentially expressed proteins in nintedanib-treated mice in cluster 2 compared to vehicle-treated mice. The list includes all identified 7006 proteins. Only proteins with $|log_2FC|>0.3$ and $p<0.05$ were considered for analysis. Each entry is described by log_2FC, confidence intervals, average expression, t-value, p-value, B-value, Entrez ID, and gene symbol. Data provided in supplemental tables file.

Supplementary Table 3. Differentially expressed proteins in nintedanib-treated mice in cluster 1 compared to nintedanib-treated mice in cluster 2. The list includes all identified 7006 proteins. Only proteins with $|log_2FC|>0.3$ and $p<0.05$ were considered for analysis. Each entry is described by log_2FC, confidence intervals, average expression, t-value, p-value, B-value, Entrez ID, and gene symbol. Data provided in supplemental tables file.

Supplementary Table 4. Results of Gene Ontology enrichment analysis of differentially expressed, downregulated proteins ($log_2FC<-0.3$, $p<0.05$) of nintedanib-treated mice in cluster 1 compared to nintedanib-treated mice in cluster 2. Each entry is described by ontology (BP, CC, or MF), GO identifier, pathway description, GeneRatio, BgRatio, p-value, FDR-adjusted p-value, q-value, and gene symbols. Data provided in supplemental tables file.

Supplementary Table 5. Results of Gene Ontology enrichment analysis of differentially expressed, upregulated proteins ($log_2FC>0.3$, $p<0.05$) of nintedanib-treated mice in cluster 1 compared to nintedanib-treated mice in cluster 2. Each entry is described by ontology (BP, CC, or MF), GO identifier, pathway description, GeneRatio, BgRatio, p-value, FDR-adjusted p-value, q-value, and gene symbols. Data provided in supplemental tables file.

Supplementary Table 6. Result of Reactome enrichment analysis of the 54 radioproteomic association modules separated by annotation of proteins positively (Spearman's $\rho\geq0.6$, $p<0.05$) and proteins negatively (Spearman's $\rho\leq-0.6$, $p<0.05$) correlating with the preclinical response-defining delta radiomic feature. Each entry is described by delta radiomic feature name, protein subset entered into enrichment analysis, Reactome identifier, pathway description, GeneRatio, BgRatio, p-value, FDR-adjusted p-value, q-value, gene symbols, and count. Data provided in supplemental tables file.

Supplementary Table 7. Result of Gene Ontology - Biological Process (GO:BP) enrichment analysis of the 54 radioproteomic association modules separated by annotation of proteins positively (Spearman's $\rho\geq0.6$, $p<0.05$) and proteins negatively (Spearman's $\rho\leq-0.6$, $p<0.05$) correlating with the preclinical response-defining delta radiomic feature. Each entry is described by delta radiomic feature name, protein subset entered into enrichment analysis, GO identifier, pathway description, GeneRatio, BgRatio, p-value, FDR-adjusted p-value, q-value, gene symbols, and count. Data provided in supplemental tables file.

Supplementary Table 8. Results of cell type enrichment analysis of the 54 radioproteomic association modules. Proteins correlating (Spearman's $|\rho|\geq0.6$, $p<0.05$) with delta radiomic features were ranked by log10 p-value and weighted by correlation coefficient prior to entering into deconvolution analysis.

Each entry is described by delta radiomic feature name, p-value, fold change difference, signed log10 enrichment p-value, cell type, and enrichment trend. Data provided in supplemental tables file.

Supplementary Table 9. Associations of patients' groups resulting from unsupervised clustering on preclinical treatment response-defining delta radiomic features (*n*=54) with clinical parameters.

	Cluster			p-value		
**Characteristic**	**A1** (*n*=3)	**A2** (*n*=10)	**A3** (*n*=6)	A1 vs. A2	A1 vs. A3	A2 vs. A3
**Baseline**						
**Age** (years)*	55.8 (±15.8)	66.5 (±13.5)	58.6 (±5.5)	0.573	0.905	0.181
**Sex**				>0.999	>0.999	>0.999
Female	0 (0%)	2 (20%)	1 (17%)			
Male	3 (100%)	8 (80%)	5 (83%)			
**Etiology**				0.7063	>0.999	0.1706
IPF	2 (67%)	6 (60%)	3 (50%)			
SSc-ILD	1 (33%)	1 (10%)	2 (33%)			
HP	0 (0%)	3 (30%)	0 (0%)			
Drug-induced ILD	0 (0%)	0 (0%)	1 (17%)			
**Disease duration** (months)†	7.2 (±5.3)	9.6 (±32.2)	38.4 (±64.6)	0.937	0.381	0.492
**Smoking status**				>0.999	>0.999	0.7902
Never	1 (33%)	2 (20%)	3 (50%)			
Previous	2 (67%)	7 (70%)	3 (50%)			
Current	0 (0%)	1 (10%)	0 (0%)			
**Pulmonary hypertension‡**				0.4965	0.500	>0.999
Yes	0 (0%)	4 (40%)	2 (33%)			
No	3 (100%)	6 (60%)	4 (67%)			
**Immunomodulatory therapy** (concomitant)§				>0.999	>0.999	0.5879
Yes	1 (33%)	4 (40%)	1 (17%)			
No	2 (67%)	6 (60%)	5 (83%)			
**FVC** (% pred)	71.0 (±28.5)	70.0 (±14.5)	50.0 (±44.5)	0.866	0.905	0.313
NA	0 (0%)	0 (0%)	0 (0%)			
**FVC** (liters)	3.010 (±0.920)	2.640 (±0.475)	2.015 (±2.310)	0.937	>0.999	0.368
NA	0 (0%)	0 (0%)	0 (0%)			
**FEV_1** (% pred)	58.0 (±22.0)	73.0 (±11.0)	52.5 (±45.8)	0.758	0.857	0.278
NA	1 (0%)	0 (0%)	0 (0%)			
**D_{LCO}** (% pred)	44.0 (±6.0)	52.5 (±11.9)	45.0 (±10.0)	0.236	0.844	0.243
NA	1 (0%)	0 (0%)	1 (0%)			
**Delta**						
**FVC** (% pred)	1.0 (±2.5)	-7.0 (±10.0)	-9.0 (3.5)	0.090	**0.024**	0.624
NA	0 (0%)	0 (0%)	0 (0%)			
**FVC** (liters)	-0.050 (±0.125)	-0.280 (±0.450)	-0.465 (±0.225)	0.112	**0.024**	0.329
NA	0 (0%)	0 (0%)	0 (0%)			
**FEV_1** (% pred)	1.5 (±4.5)	-4.0 (±6.8)	-9.0 (±10.0)	0.286	0.118	0.200
NA	1 (0%)	0 (0%)	1 (0%)			
**D_{LCO}** (% pred)	3.0 (±8.0)	-6.0 (±11.0)	-13.0 (±10.0)	0.381	0.267	0.461
NA	1 (0%)	5 (0%)	2 (0%)			

Data are presented as median (± interquartile range (IQR)) or n (%). Mann-Whitney U and Fisher's exact tests were used to compare the numerical and categorical variables, respectively. Abbreviations: IPF, idiopathic

pulmonary fibrosis; ILD, interstitial lung disease; SSc, systemic sclerosis; HP, hypersensitivity pneumonitis; FVC, forced vital capacity; FEV1, forced expiratory volume in 1 s; DLCO, diffusing capacity of the lung for carbon monoxide; P(A)H, pulmonary (arterial) hypertension. NA denotes missing values as n (%). *: Age at time of baseline (pre-treatment) HRCT scan. †: disease duration was defined as the period (months) between first reported diagnosis of PF-ILD in the patient records and the baseline (pre-treatment) HRCT scan. ‡: PH was assessed by echocardiography or right heart catheterization. If right heart catheterization was performed, mPAP>20 mmHg was considered diagnostic (1). §: Concomitant immunomodulatory therapy was indicated if a patient received immunomodulatory medication at any time simultaneous to nintedanib treatment. Immunomodulatory therapy included prednisolone, mycophenolate mofetil, azathioprine, rituximab, tocilizumab, or combinations thereof.

Supplementary Table 10. Associations of patients' groups resulting from unsupervised clustering on ECM remodeling-associated delta radiomic features (*n*=8) with clinical parameters.

	**Cluster**		**p-value**
**Characteristic**	**B1** (*n*=11)	**B2** (*n*=8)	B1 vs. B2
**Baseline**			
**Age** (years)*	58.3 (±16.1)	63.2 (±7.6)	0.717
**Sex**			
Female	2 (18%)	1 (12%)	>0.999
Male	9 (82%)	7 (88%)	
**Etiology**			
IPF	7 (64%)	4 (50%)	
SSc-ILD	2 (18%)	2 (26%)	0.890
HP	2 (18%)	1 (12%)	
Drug-induced ILD	0 (0%)	1 (12%)	
**Disease duration** (months)†	12.0 (±32.2)	11.1 (±50.5)	0.717
**Smoking status**			
Never	3 (27%)	3 (38%)	
Previous	7 (64%)	5 (62%)	>0.999
Current	1 (9%)	0 (0%)	
**Pulmonary hypertension‡**			
Yes	2 (18%)	4 (50%)	0.3189
No	9 (82%)	4 (50%)	
**Immunomodulatory therapy§**			
Yes	3 (27%)	3 (38%)	>0.999
No	8 (73%)	5 (62%)	
**FVC** (% pred)	69.0 (±18.0)	64.5 (±37.0)	>0.999
NA	0 (0%)	0 (0%)	
**FVC** (liters)	2.630 (±0.845)	2.665 (±1.248)	0.778
NA	0 (0%)	0 (0%)	
**FEV_1** (% pred)	71.0 (±23.8)	69.0 (±40.0)	0.859
NA	1 (9%)	0 (0%)	
**D_{LCO}** (% pred)	50.8 (±10.8)	45.0 (±22.0)	0.557
NA	1 (9%)	1 (12%)	
**Delta**			
**FVC** (% pred)	-4.0 (±5.5)	-10.5 (±5.5)	**0.047**
NA	0 (0%)	0 (0%)	
**FVC** (liters)	-0.200 (±0.205)	-0.530 (±0.245)	**0.035**
NA	0 (0%)	0 (0%)	
**FEV_1** (% pred)	-3.0 (±3.0)	-9.0 (±7.5)	0.055
NA	1 (9%)	1 (12%)	
**D_{LCO}** (% pred)	-5.5 (±14.8)	-9.0 (±7.0)	>0.999
NA	3 (27%)	5 (50%)	

Data are presented as median (± interquartile range (IQR)) or n (%). Mann-Whitney U and Fisher's exact tests were used to compare the numerical and categorical variables, respectively. Abbreviations: IPF, idiopathic pulmonary fibrosis; ILD, interstitial lung disease; SSc, systemic sclerosis; HP, hypersensitivity pneumonitis; FVC, forced vital capacity; FEV1, forced expiratory volume in 1 s; DLCO, diffusing capacity of the lung for carbon monoxide; P(A)H, pulmonary (arterial) hypertension. NA denotes missing values as n (%). *: Age at time of baseline (pre-treatment) HRCT scan. †: disease duration was defined as the period (months) between first reported diagnosis of PF-ILD in the patient records and the baseline (pre-treatment) HRCT scan. ‡: PH was assessed by echocardiography or right heart catheterization. If right heart catheterization was performed, mPAP>20 mmHg was considered diagnostic (1). §: Concomitant immunomodulatory therapy was indicated if a patient received immunomodulatory medication at any time simultaneous to nintedanib treatment. Immunomodulatory therapy included prednisolone, mycophenolate mofetil, azathioprine, rituximab, tocilizumab, or combinations thereof.

Supplementary Table 11. Summary of HRCT scan acquisition parameters.

CT parameter	Description
Manufacturer(s)*	Siemens, Siemens Healthineers, Philipps, Toshiba
Acquisition mode	inspiration (breath hold)
Position	head first-supine (HFS), feet first-supine (FFS)
Slice thickness (mm)	1 [range 0.5-1.5]
Reconstruction kernels	I70f, I80s, Br56f, Br56u, Br59f, LUNG, FC55 (sharp), YB
Tube voltage (kVp)	120 (range 80-130)

*HRCT scanner models included SOMATOM Definition Flash (Siemens), SOMATOM Definition Edge (Siemens), SOMATOM Scope (Siemens) NAEOTOM Alpha (Siemens Healthineers), Acquilion (Toshiba), Brilliance 64 (Philips).

Supplementary Table 12. Mouse primer sequences used for quantitative PCR reactions.

Gene	Forward primer (5' → 3')	Reverse primer (5' → 3')
Ccl2	CCA CTC ACC TGC TGC TAC TCA T	TGG TGA TCC TCT TGT AGC TCT CC
Cd40lg	CAC ACG TTG TAA GCG AAG CC	ACC GTC AGC TGT TTC CCA TT
Col1a1	GAT GAC GTG CAA TGC AAT GAA	CCC TCG ACT CCT ACA TCT TCT GA
Col3a1	AGC TTT GTG CAA AGT GGA ACC	ATA GGA CTG ACC AAG GTG GC
Cxcl1	ACT CAA GAA TGG TCG CGA GG	GTG CCA TCA GAG CAG TCT GT
Fn1	ATG TGG ACC CCT CCT GAT AGT	GCC CAG TGA TTT CAG CAA AGG
Il1b	TGC CAC CTT TTG ACA GTG ATG	TGA TGT GCT GCT GCG AGA TT
Il6	TGA TGG ATG CTA CCA AAC TGG	GGT ACT CCA GAA GAC CAG AG
Rplp0	GCA GGT GTT TGA CAA CGG CAG	GAT GAT GGA GTG TGG CAC CGA
Spp1	AGT GAC TGA TTC TGG CAG CTC	ATC TGG GTG CAG GCT GTA AA
Tgfb1	CTG GAG TTG TAC GGC AGT GG	GTT CAT GTC ATG GAT GGT GCC
Timp1	GGC ATC TGG CAT CCT CTT GT	CGC TGG TAT AAG GTG GTC TCG
Tnf	ACC ACG CTC TTC TGT CTA CTG	ACT GAT GAG AGG GAG GCC ATT

Supplementary Table 13. Primary and secondary antibodies used for immunofluorescence staining.

Description	Target	Reactivity	Host	Company	Label	Catalog / Clone	Dilution
Primary Ab	α-SMA	ms (hu, rt)	ms	Sigma-Aldrich	none	A5228 (1A4)	1:1500
Primary Ab	proSP-C	ms (hu, rt)	rb	Sigma-Aldrich	none	AB3786 (pAb)	1:200
Primary Ab	PDPN	ms	gt	R&D Systems	none	AF3244 (pAb)	1:200
Secondary Ab	IgG	ms	dk	Invitrogen	Alexa488	A21202 (pAb)	1:250
Secondary Ab	IgG	rb	dk	Invitrogen	Alexa568	A10042 (pAb)	1:250
Secondary Ab	IgG	gt	dk	Invitrogen	Alexa647	A21447 (pAb)	1:250

Abbreviations: Ab, antibody; pAb, polyclonal antibody; IgG, immunoglobulin G; ms, mouse; hu, human; rt, rat; rb, rabbit; gt, goat; dk, donkey; α-SMA, alpha-smooth muscle actin; proSP-C, prosurfactant protein C; PDPN, podoplanin.

Supplementary Table 14. Variable importance selected delta radiomic features used to classify treatment response.

#	Feature name	Class	Subclass	Filter	Score
1	*GLSZM_smallZone_lowGL_emp*	Texture	Texture	none	0.92
2	*LHL_GLSZM_GLvar*	Wavelet	Texture-derived	LHL	0.96
3	*center_mass_shift*	Shape	Shape	none	1.00
4	*LLL_hist_skewness*	Wavelet	Intensity-derived	LLL	1.00
5	*HLH_mGLCM_clust_prominence*	Wavelet	Texture-derived	HLH	0.92
6	*LHL_GLRLM_entropy*	Wavelet	Texture-derived	LHL	0.92
7	*HHL_GLRLM_runPercentage*	Wavelet	Texture-derived	HHL	0.92
8	*LHH_GLRLM_shortRunEmp*	Wavelet	Texture-derived	LHH	1.00
9	*LLH_mGLCM_IMC1*	Wavelet	Texture-derived	LLH	0.96
10	*HLL_hist_energy*	Wavelet	Intensity-derived	HLL	0.96
11	*LHH_mGLRLM_runPercentage*	Wavelet	Texture-derived	LHH	0.92
12	*LLL_GLSZM_largeZone_highGL_emp*	Wavelet	Texture-derived	LLL	0.92
13	*LHL_mGLCM_IMC2*	Wavelet	Texture-derived	LHL	1.00
14	*LHL_mGLCM_IMC1*	Wavelet	Texture-derived	LHL	1.00
15	*HLH_GLRLM_entropy*	Wavelet	Texture-derived	HLH	0.96
16	*LLH_mGLCM_MCC*	Wavelet	Texture-derived	LLH	0.92
17	*LLH_mGLCM_clust_prominence*	Wavelet	Texture-derived	LLH	1.00
18	*HLL_mGLCM_correlation*	Wavelet	Texture-derived	HLL	0.96
19	*HLH_hist_energy*	Wavelet	Intensity-derived	HLH	0.92
20	*LHL_mGLCM_MCC*	Wavelet	Texture-derived	LHL	1.00
21	*LLL_hist_coeffOfVar*	Wavelet	Intensity-derived	LLL	1.00
22	*HHL_mGLCM_IMC1*	Wavelet	Texture-derived	HHL	0.92
23	*LLH_GLSZM_ZSnonuniformity*	Wavelet	Texture-derived	LLH	0.96
24	*LLL_GLSZM_GLvar*	Wavelet	Texture-derived	LLL	0.96
25	*LHL_GLDZM_ZSnonuniformity_norm*	Wavelet	Texture-derived	LHL	0.92
26	*LHL_mGLCM_homogeneity_norm*	Wavelet	Texture-derived	LHL	0.92
27	*LLH_hist_energy*	Wavelet	Intensity-derived	LLH	1.00
28	*LLL_hist_kurtosis*	Wavelet	Intensity-derived	LLL	0.92
29	*LLH_GLSZM_GLnonuniformity_norm*	Wavelet	Texture-derived	LLH	1.00
30	*LLL_hist_percentile90*	Wavelet	Intensity-derived	LLL	0.92

31	*LHH_mGLCM_IMC2*	Wavelet	Texture-derived	LHH	1.00
32	*LHL_GLCM_MCC*	Wavelet	Texture-derived	LHL	0.92
33	*HHH_GLCM_IMC1*	Wavelet	Texture-derived	HHH	1.00
34	*HHH_GLRLM_LRvar*	Wavelet	Texture-derived	HHH	1.00
35	*GLSZM_smallZoneEmp*	Texture	Texture	none	0.98
36	*LHH_mGLCM_IMC1*	Wavelet	Texture-derived	LHH	1.00
37	*HHH_mGLRLM_entropy*	Wavelet	Texture-derived	HHH	1.00
38	*LHL_hist_energy*	Wavelet	Intensity-derived	LHL	1.00
39	*LHH_mGLRLM_GLnonuniformity_norm*	Wavelet	Texture-derived	LHH	1.00
40	*HHL_mGLRLM_entropy*	Wavelet	Texture-derived	HHL	0.96
41	*LHL_NGTDM_contrast*	Wavelet	Texture-derived	LHL	0.92
42	*LLH_GLCM_MCC*	Wavelet	Texture-derived	LLH	0.92
43	*LHL_GLSZM_ZSnonuniformity*	Wavelet	Texture-derived	LHL	0.96
44	*HHH_NGTDM_busyness*	Wavelet	Texture-derived	HHH	1.00
45	*HHH_mGLCM_MCC*	Wavelet	Texture-derived	HHH	1.00
46	*HLH_GLSZM_ZSnonuniformity*	Wavelet	Texture-derived	HLH	1.00
47	*LHH_GLCM_IMC1*	Wavelet	Texture-derived	LHH	0.94
48	*LHL_GLDZM_ZSnonuniformity*	Wavelet	Texture-derived	LHL	1.00
49	*HHH_GLRLM_longRun_lowGL_emp*	Wavelet	Texture-derived	HHH	0.96
50	*HHH_NGTDM_strength*	Wavelet	Texture-derived	HHH	1.00
51	*HHH_NGTDM_complexity*	Wavelet	Texture-derived	HHH	1.00
52	*LLH_GLSZM_entropy*	Wavelet	Texture-derived	LLH	0.92
53	*HHH_GLDZM_largeDistance_highGL_emp*	Wavelet	Texture-derived	HHH	0.92
54	*LHH_GLCM_MCC*	Wavelet	Texture-derived	LHH	0.96

Abbreviations: GLCM = Gray Level Co-occurrence Matrix, NGTDM = Neighborhood Gray Tone Difference Matrix, GLRLM = Gray Level Run Length Matrix, GLDZM = Gray Level Distance Matrix and NGLDM = Neighboring Gray Level Dependence Matrix.

Supplementary Methods

<u>Animal experimentation and ethics statement</u>

The effects of antifibrotic treatment on delta radiomics were studied in mice with bleomycin-induced lung fibrosis (2–5). To induce lung fibrosis, C57BL/6J-Rj mice (*n*=30, female, 8-weeks old, Janvier Labs, Le Genest-Saint-Isle, France) were intratracheally instilled with 2 U/kg bleomycin sulfate (Bleomycin Baxter 15'000 I.U., pharmacy of the canton Zurich, Switzerland) dissolved in saline on day 0. Treatment with 60 mg/kg nintedanib (*n*=15) or vehicle-only (deionized water, *n*=15) was administered once daily by oral gavage (at a volume of 10 μL/g body weight) from day 7 to day 20 for a total of 14 applications. Lung microCT scans were acquired of each animal pre- (day 7) and post-treatment (day 21) for generation of radiomic feature sets. All mice were sacrificed on day 21 by CO_2 inhalation (24 hours after the final treatment), followed by exsanguination of the vena cava and transcardial perfusion of the lungs with 5-10 mL ice-cold Dulbecco's phosphate-buffered saline (DPBS) at a pressure of 100-120 cm H_2O to remove residual blood from the lung. The lung was excised, rinsed with DPBS, dissected into the individual lobes, and processed according to the different assay requirements. Mice were allocated to different study groups by complete randomization and treatment was provided in a double-blinded manner. All mice were housed in groups of five with access to food and water *ad libitum* in standard housing conditions with 12-hour light-dark cycles. Animals were acclimatized for seven days prior to experimentation start. HydroGel® (ClearH2O Inc.) and water-soaked standard rodent diet were provided to alleviate body weight loss. Paracetamol analgesia was provided if mice showed signs of pain. Ethical approval for experimentation was granted by the cantonal veterinary office (license no. ZH082/2021) and experimentation was performed in strict compliance with Swiss animal protection laws and guidelines. Mice were excluded from analyses if humane endpoints were reached (*n*=3) or if microCT scans exhibited presence of severe lung abnormalities (*n*=3), including atelectasis or unilateral fibrosis development. Nintedanib esilate compound for use in this study was provided by Dr. Lutz Wollin (Boehringer Ingelheim Pharma GmbH & Co. KG, Biberach an der Riss, Germany).

<u>Patient cohort, clinical data, and ethics statement</u>

In this study, 19 PF-ILD (6, 7) patients undergoing treatment with nintedanib at Bern University Hospital were retrospectively selected from the Bern University Hospital registry and the SWISS-IIP cohort. Approval for the study was granted by the local ethics committee (BASEC-ID: 2023-01920 [ILDALMO]; PB_2016_01524 [SWISS-IIP cohort]). Selection was performed based on the inclusion and exclusion criteria stated below. A total of 359 patients diagnosed with (progressive) fibrosing ILD were screened for fulfillment of the below criteria, of which 54 patients received nintedanib treatment for ≥6 months. Of these 54 patients, 19 also fulfilled the remaining inclusion criteria.

Inclusion criteria

1. Diagnosis of PF-ILD determined by a senior attending physician according to established guidelines (8, 9)
2. Treatment with nintedanib (≥100 mg twice daily; min. 6 months at follow-up HRCT)
3. Availability of HRCT chest scans fulfilling the following criteria:
    a. Pre-treatment HRCT (max. 1 month after treatment initiation)
    b. Post-treatment HRCT (min. 6 months interval to pre-treatment HRCT)
    c. Slice thickness in range 0.5 - 1.5 mm
    d. Acquisition at tube voltage in range 80-130 kVp
    e. One of the following reconstruction kernels: I70f, I80s, Br56f, Br56u, Br59f, LUNG, FC55 (sharp), YB
    f. Filtered-back projection as reconstruction algorithm
    g. Scans acquired in full inspiration mode
4. Availability of PFT recordings fulfilling the following criteria:

a. Pre-treatment PFT (max. 1 month after treatment initiation)
b. Post-treatment PFT (min. 6 months interval to pre-treatment PFT)

Exclusion Criteria

5. Presence of secondary lung disease at times any HRCT scans and PFT recording (e.g., cancer, COVID-19, pneumonia, bronchitis)

Demographic and clinical parameters were derived from electronic patient records, including age (birth date), sex, disease etiology, date of diagnosis, date of nintedanib treatment start, presence of pulmonary (arterial) hypertension, smoking status, concomitant medications, and dates of PFT and HRCT scan recordings. The recorded PFT parameters included forced vital capacity (FVC) in % pred. and liters. Changes in PFT recordings between pre- and post-treatment were expressed as delta values. A Summary of patient demographics and clinical characteristics is provided in **Table 1**.

MicroCT image acquisition

Lung microCT images of each mouse were acquired at days 7 and 21 on a SkyScan 1176 (Bruker, Kontich, Belgium) in free-breathing conditions under isoflurane anesthesia using respiratory gated image acquisition. Anesthesia was induced by 5.0% and maintained by 1.5-2.5% isoflurane in air at 0.8-1.0 L/min flow rate to achieve a breathing rate of 0.7-0.9 breaths/s for an average scan time of 15 min. Animals were placed in supine position on the scanner bed with a styrofoam block mounted on the diaphragm to allow monitoring of respiratory gating. Image acquisition was performed with the following acquisition settings: tube voltage = 50 kV, tube current = 500 μA, filter = Al 0.5 mm, frame averaging = on (3), rotation step = 0.7 degrees, sync with events = 50 ms, X-ray tube rotation = 360 degrees, exposure time = 77 ms, resolution = 35 μm, slice thickness = 35 μm. Images were reconstructed with NRecon software (v.1.6.8.0; Bruker) using Feldkamp filtered back-projection algorithm with the following parameters: misalignment compensation (scan-dependent manual adjustment), smoothing = 1 with Gaussian kernel, ring artifact compensation = 4, and beam hardening correction = 10%. Reconstructed images were converted to DICOM format.

CT segmentation of mouse lungs

Left and right lung lobes of mice were semi-automatically segmented by two readers (D.L., M.B.) using MIM software (v.7.1.6, MIM Software Inc., Cleveland, Ohio, USA). Briefly, a seed was set within the right and left lung using the "region grow" tool (upper limit = -600 HU, lower limit = -800 HU, tendril diameter = 0.2 mm, fill holes = strong), which then automatically defined the vast majority of the lung volume in the 3D space. Manual contour alignment with the 2D/3D brush was used to correct misaligned areas. Finally, the smoothing function was used to remove sharp edges. For medical diagnostics, the Hounsfield scale is usually normalized to 120 kVp tube voltage, which could technically not be achieved by our microCT instrument. To enable direct comparison between CT-derived radiomic datasets from patients and mice, the reconstructed microCT images of mouse lungs were pixel value corrected to match clinical specifications as previously described (10).

HRCT image acquisition

HRCT acquisition of human lungs was performed at Bern University Hospital or outpatient clinics. Instrument and scan settings used are summarized in **Supplementary Table 11**. All HRCT scans were evaluated by a senior radiologist (L.E.) at the Department of Diagnostic, Interventional, and Pediatric Radiology of the Bern University Hospital for the presence of PF-ILD on a standard picture archiving and communication system workstation and a radiology-grade display monitor.

CT segmentation of human lungs

Left and right lung lobes were semi-automatically segmented by two readers (C.M., L.K.) with the open-source software 3D Slicer (v.5.2.1). Pulmonary hilar vessels and atelectatic areas were manually

excluded from the regions of interest. Manual contour corrections were only applied when spatially limited areas did not coincide with the actual borders of the lungs.

Pulmonary function tests

Pulmonary function tests were performed by trained personnel at the Department of Pulmonary Medicine of the Bern University Hospital or in outpatient clinics. All tests were performed following established protocols (11–14).

Radiomic feature calculation

Calculation of radiomic features was performed on merged structures of left and right lung lobes using Z-Rad software (v.7.3.1, https://medical-physics-usz.github.io/, Department of Medical Physics, University Hospital Zurich, Zurich, Switzerland), an image biomarker standardization initiative (IBSI)-compliant Python-based software (15), as described in (10). Mouse lungs were resized to isotropic voxels of 0.15 mm. To achieve comparable voxel size in patients, human lungs were resized to isotropic voxels of 2.75 mm, corresponding to an estimated 6000-fold volumetric difference (16). Both mouse and human lung volumes were discretized to a fixed bin size of 50 HU in a range of -1000 HU to 200 HU. From the resized volumes, 1388 radiomic features were calculated per lung scan and time point (HU limits: -1000 to 200 HU), corresponding to the following feature classes:

1. Histogram features (*n*=17)
2. Texture features (*n*=137): Gray Level Co-occurrence Matrix (*n*=52, GLCM), Neighborhood Gray Tone Difference Matrix (*n*=5, NGTDM), Gray Level Run Length Matrix (*n*=32, GLRLM), Gray Level Size Zone Matrix (*n*=16, GLSZM), Gray Level Distance Matrix (*n*=16, GLDZM), and Neighboring Gray Level Dependence Matrix (*n*=16, NGLDM)
3. Wavelet features (*n*=1232): Transformation of histogram and texture features following coiflet filter decomposition
4. Shape features (*n*=2)

Histogram features carry information about distribution of voxel intensities using first-order statistics (e.g. mean, standard deviation, skewness, kurtosis), describing tissue intensity characteristics. Texture features define intra-tissue heterogeneity by calculating the spatial relationship between neighboring voxel intensities (17). Wavelet features compute histogram and texture features after wavelet decompositions of the original image using eight different coiflet filters (high- to low-pass filters), thereby concentrating the features on different frequency ranges (18). Shape features describe tissue volume and size independent of intensity distribution.

Delta radiomic features describing the change of each feature between pre-and post-treatment were expressed as delta values: ΔFeature = Feature (t_2) – Feature (t_1) (19). Lung densitometric information was directly inferred from the radiomic histogram feature *hist_mean*, which describes the lung attenuation-based average HU intensity of the segmented lung volume.

Radiomic feature stability evaluation

Intraclass correlation coefficients (ICC) were calculated for each radiomic feature to evaluate stability against inter- and intra-reader bias in the lung segmentation process. For inter- and intra-reader ICC, two (D.L., M.B.) and one examiner(s) (D.L.), respectively, independently segmented 16 randomly selected mouse lung scans, followed by radiomic feature calculation of the delineation structures. ICCs were calculated using two-way mixed effect models with the *consistency* method in the *irr* R package according to published reports (20). Only stable/reproducible features (*n*=1130) with ICC≥0.75 were considered for further analyses for both mouse and human datasets (21). Feature stability assessment was performed on the mouse dataset due to the lesser degree of automation in lung segmentation.

Proteomics

For comparative proteomics, the middle lobe of the right mouse lung was snap frozen in liquid nitrogen and stored at -80°C until processing. Sample workup and data collection was performed by trained personnel at the Proteomics and Mass Spectrometry Core Facility (PMSCF) at the University of Bern using standard established protocols. All vehicle- (*n*=14) and nintedanib-treated (*n*=10) samples were analyzed. One vehicle sample was excluded from analysis due sample workup issues. Tissue homogenization was performed in 8M urea / 100 mM Tris (pH 8.0) buffer supplemented with *cOmplete* protease inhibitor cocktail (Roche Diagnostics, Mannheim, Germany) using the FastPrep system (MP Biomedicals). Following reduction, alkylation, and overnight protein precipitation with ice-cold acetone, 10 μg of the cleaned protein mixture was digested into peptides using a two-step digestion protocol (LysC for 2 h at 37°C followed by Trypsin at room temperature overnight). Digests were analyzed by nano-liquid chromatography on a Dionex Ultimate 3000 (ThermoFisher Scientific, Reinach, Switzerland) through a CaptiveSpray source (Bruker, Bremen, Germany) with an end-plate offset of 500 V, a drying temperature of 200°C, and with the capillary voltage fixed at 1.6 kV. A volume of 2 μL (200 ng) protein digest was loaded onto a pre-column (PepMap 100 C18, 5 μm, 100 A, 300 μm diameter x 5 mm length, ThermoFisher) at a flow rate of 10 μL/min with 0.05% trifluoroacetic acid in water / acetonitrile 98:2. After loading, peptides were eluted in back flush mode onto an in-house made C18 CSH Waters column (1.7 μm, 130 Å, 75 μm x 20 cm) by applying a 90-minute gradient of 5% acetonitrile to 40% in water / 0.1% formic acid, at a flow rate of 250 nL/min. The timsTOF Pro instrument (Bruker, Bremen, Germany) was operated either in data-dependent acquisition (DDA) or data-independent (DIA) mode using the Parallel Acquisition Serial Fragmentation (PASEF) option. The mass range was set between 100 and 1700 m/z, with 10 PASEF scans between 0.7 and 1.4 V s/cm^2. The accumulation time was set to 2 ms, and the ramp time was set to 100 ms, respectively. Fragmentation was triggered at 20’000 arbitrary units, and peptides (up to charge of 5) were fragmented using collision induced dissociation with a spread between 20 and 59 eV. DDA data was processed further with FragPipe software (v.17.0) using the IonQuant algorithm and filtering protein identifications to a 1% false discovery rate (FDR) on the peptide level using the Percolator algorithm. Furthermore, protein groups were filtered by the criterion that at least two different razor peptide sequences were identified as evidence for the existence of the protein group. From the DDA data, a spectral library was built with the FragPipe software. This library was used to identify and quantify proteins with the DIA data using standard parameters in Spectronaut 16 software (Biognosys, Schlieren, Switzerland). Protein names (Uniprot IDs) were converted to Entrez IDs and Gene Symbols using *UniProt.ws* and *annotationDbi* R packages. Protein names without matching Entrez Gene ID were dropped, resulting in a final set of 7006 proteins.

Phosphoproteomics

For phosphoproteomics, the middle lobe of the right mouse lung was snap frozen in liquid nitrogen after collection and stored at -80°C until processing. Sample workup and data pre-processing was performed by trained personnel at the Proteomics and Mass Spectrometry Core Facility (PMSCF) at the University of Bern using standard established protocols. Randomly selected subsets of vehicle- (*n*=5) and nintedanib-treated (*n*=5) were analyzed. A titanium dioxide phosphopeptide enrichment workflow (22) with subsequent DDA liquid chromatography tandem mass spectrometry (LC-MS) analysis on the same instrument and parameter settings as described above was applied. Samples were searched and quantified with FragPipe (23) (v.18.0, MSFragger version 3.5, Philosopher version 4.4.0, IonQuant version 1.8.0) using the following parameters: swissprot (24) Mus musculus database (release 2022_01) with isoforms and common contaminants; 20 ppm and 0.05 Da mass tolerance for precursors and fragment, respectively; search enzyme trypsin with max 3 allowed missed cleavages; fix modification: carbamidomethylation of cysteine; variable modifications (altogether max 4/peptide): methionine oxidation of methionine (max 3/peptide), phosphorylation of serine, threonine and tyrosine (max 3/peptide) and protein N-terminal acetylation. Peptide forms normalized with the variance

stabilization (25) normalization method are reported as NormI, along FragPipe's MaxLFQ and IonQuant's FragI abundance measures. The intensities of peptide forms were combined as protein phosphosite locations by summing the corresponding contributions.

Kinase activity enrichment analysis

Differential expression of phosphosites and subsequent kinase activity enrichment analysis was performed as described in (22). First, the phosphosites' missing values were imputed using a left-censored Gaussian replacement method if there was more than 1 missing value in a group of replicate, and a maximum likelihood estimation otherwise (26). A moderated t-statistic (26) was then calculated for each phosphosite, and used as the ranking metric for the Kinase Activity Enrichment Analysis (KAEA) tool (22). KAEA was then applied on the ranked phosphosite list and reversed ranked list using the included mouse kinase substrate database. SetRank set p-value and FDR cutoff were set to 0.01 and 0.05, respectively.

Differential protein expression analysis

Differential expression of proteins between groups of interest was calculated in R using the "limma" package according to standard guidelines (27). At first, DIA-based Spectronaut protein expression intensities were log_2-transformed. Then, log_2 fold changes were calculated as contrasts by application of a linear model using robust regression for each protein. Finally, estimated coefficients and standard errors for the given set of contrasts were calculated for each protein, followed by Empirical Bayes smoothing of standard errors. Proteins with $log_2FC>0.3$ ($p<0.05$), corresponding to 23% mean expression change, were considered as statistically significant.

Gene expression analysis

RNA was isolated from blood-free cranial lobes of the right mouse lung stored in RNAlater (ThermoFisher Scientific). Tissues were mechanically homogenized with the TissueLyser II instrument (Qiagen, Hombrechtikon, Switzerland), followed by total RNA isolation with the RNeasy Tissue Mini Kit (Qiagen, Hombrechtikon, Switzerland). Isolated RNA was reverse transcribed into cDNA using the Transcriptor First Strand cDNA Synthesis Kit (Roche Diagnostics, Switzerland). Expression of fibrotic (*Col1a1*, *Col3a1*, *Fn1*), inflammatory (*Il6, Ccl2*, *Spp1*), and nintedanib-related (*Tgfb1*, *Timp1*, *Cxcl1*, *Ifng*, *Il1b*, *Tnf*, *Cd40lg*) genes was analyzed by SYBR Green quantitative PCR using GoTaq Green Master Mix kit (Promega) as described in (28). Expression of mRNA was expressed to delta Ct values (Ct [gene of interest] – Ct [reference gene]) with *Rplp0* as reference gene. Lower delta Ct values indicate higher target gene expression. Fold changes relative to vehicle-treated samples were calculated using the delta-delta Ct method. The list of the primer pairs used in this study is provided in **Supplementary Table 12**.

Immunofluorescence and microscopy

Formalin-fixed paraffin-embedded lung sections (3 μm thickness) were cut on a HistoCore Multicut microtome (Biosystems Switzerland AG, Muttenz, Switzerland). Following deparaffinization, heat-mediated antigen retrieval with R-Universal Buffer (Cat. AP0530-500, Aptum Biologics) was performed for 15 min at 95°C. After incubation for 25 min at RT for cooling, blocking of unspecific antibody staining was performed with 5% BSA in antibody diluent (Cat. S3022, Dako) for 1 h at RT. Primary antibodies dissolved in antibody diluent (Cat. S3022, Dako) were then applied and incubated overnight at 4°C. Next, samples were incubated with secondary antibodies dissolved in PBS supplemented with 1% BSA for 2 h at RT. All antibodies and the dilutions used are listed in **Supplementary Table 13**. Finally, cell nuclei were counterstained with 4',6-diamidino-2-phenylindole (DAPI) for 10 min at RT. The sections were then scanned in immunofluorescence mode on a AxioScan.Z1 slide scanner (Zeiss, Feldbach, Switzerland) using a Plan-Apochromat 20x/0.8 M27 objective. Cells positively stained for α-SMA were quantified using the "Positive cell detection" tool of the open source software QuPath (v.0.4.0) at default

parameter settings and detection thresholds 800, respectively (29). From each sample, five representative areas at 500x500 μm were quantified and the average was used for statistical analyses.

Unsupervised clustering

All variables were z-scored ([x-mean]/standard deviation) prior to analysis (*clusterSim* R package). Unsupervised agglomerative hierarchical (using Euclidean distance with complete linkage method) or k-means clustering was performed to identify subgroups of mice or patients with similar delta radiomic feature patterns or proteomic profiles using base R functions. Clusterability was evaluated by Hopkin's statistic H, with H>0.5 indicating clusterability (*hopkins* R package) (30). The optimal number of clusters was determined by average silhouette statistics inspecting k clusters between 2 and 5, selecting the optimal k based on global or local optimum for separation (*factoextra* R package). Stability of clusters was assessed by Jaccard bootstrapping with n=1'000 iterations (*fpc* R package) (31).

Variable importance evaluation

To estimate the importance of each delta radiomic feature for the classification produced by unsupervised clustering, we calculated filter-based variable importance using the "caret" package (32) and retained features with classification score≥0.9. Features (*n*=54) most important for differentiating clusters 1 and 2 in nintedanib-treated mice are listed in **Supplementary Table 14**.

Gene Ontology and Reactome pathway enrichment

Curated lists of DE or highly-correlated proteins were used to perform Gene Ontology (GO) or Reactome pathway enrichment analysis using the R packages *clusterProfiler* and *ReactomePA*, respectively (33–35), retaining results after false discovery rate adjustment ($p<0.05$). In case of enrichment of proteins highly correlated with delta radiomic features, proteins with positive and negative correlation coefficients were entered separately into GO or Reactome pathway enrichment analysis. To visualize and interpret results, the GeneRatios of positive and negative enriched pathways were transformed into matrices with delta radiomic features as columns and pathways terms as rows. Then, results with GeneRatios<0.10 were dropped (set to zero), and only pathway terms enriched (GeneRatio≥0.10) in at least two delta radiomic features were retained. Subsequently, the tables containing positive and negative enriched delta radiomic pathway pairs were aggregated, and rows and columns without significantly enriched results were removed, followed by visualization with the *pheatmap* R package.

Correlation analysis

Spearman's rank correlation coefficient rho was calculated between selected delta radiomic features and the $\log_2$-transformed expression intensity of every protein using base R packages, retaining only proteins with $p<0.05$ and $\rho\geq0.6$ for further analysis. Pearson's correlation coefficient r was calculated between selected delta radiomic features and the fraction of α-SMA positive cells using base R packages.

Cell type signature enrichment analysis

To infer relative cell type frequency changes between two groups from proteomics data, we applied signature enrichment analysis as described in (36, 37), utilizing their published dataset. Cell type signatures were defined as sets of genes with cell-type specific gene expression of $\log_2$ fold change>0.3 and adjusted $p<0.05$ (37). For each cell type, we then tested for the enrichment in a ranked list of DE proteins ($\log_2$ fold changes) or correlation coefficients (weighted by $-\log_{10}$ p-value) using the Kolmogorov-Smirnov test. Positive and negative signed enrichment scores ($-\log_{10}$ p-values signed by effect size) reflect relative depletion and enrichment of the respective cell type, respectively. To visualize and interpret cell type signatures enriched in the sets of proteins highly-correlation (Spearman's $|\rho|\geq0.6$, $p<0.05$) with delta radiomic features, the signed enrichment scores of each

variable were transformed into a matrix with delta radiomic features as columns and cell types as rows. Enriched results with enrichment score<2 (corresponding to p<0.01) were dropped (set to zero), followed by removal of rows and columns without enriched entries, and visualization with the *pheatmap* R package.

Association analysis with clinical parameters

Association analyses were performed to investigate associations of patient delta radiomics-derived (k-means) clusters with clinical parameters. Mann-Whitney U test was used for comparison of numerical variables, and Fisher's exact test was used to compare categorical variables.

Statistical analyses

All statistical analyses were performed in R (v.4.3.1.) environment. For all analyses, a p<0.05 was considered statistically significant unless stated otherwise. The following R packages were used: *AnnotationDbi*, *caret*, clusterProfiler, *clusterSim*, *doParallel*, *dplyr*, *factoextra*, *fpc*, *ggplot2*, *ggpubr*, *ggrepel*, *limma*, *openxlsx*, *org.Mm.eg.db*, *parallel*, *pheatmap*, *readxl*, *rstatix*, *tidyverse*, *UniProt.ws*, *VennDiagram*.

Data Visualization

Figures were created in Adobe Illustrator (v.28.2) and partially contain graphics or illustrations from Adobe Stock and BioRender.com accessed through the academic licenses of the University of Bern.

References

1. Humbert M, et al. 2022 ESC/ERS Guidelines for the diagnosis and treatment of pulmonary hypertension. *Eur Heart J*. 2022;43(38):3618–3731.

2. Wollin L, et al. Antifibrotic and anti-inflammatory activity of the tyrosine kinase inhibitor nintedanib in experimental models of lung fibrosis. *J Pharmacol Exp Ther*. 2014;349(2):209–220.

3. Ackermann M, et al. Effects of nintedanib on the microvascular architecture in a lung fibrosis model. *Angiogenesis*. 2017;20(3):359–372.

4. Ruscitti F, et al. Quantification of Lung Fibrosis in IPF-Like Mouse Model and Pharmacological Response to Treatment by Micro-Computed Tomography. *Front Pharmacol*. 2020;11:1117.

5. Mecozzi L, et al. In-vivo lung fibrosis staging in a bleomycin-mouse model: a new micro-CT guided densitometric approach. *Sci Rep*. 2020;10(1):18735.

6. Kolb M, Vašáková M. The natural history of progressive fibrosing interstitial lung diseases. *Respir Res*. 2019;20(1):57.

7. Raghu G, et al. Idiopathic Pulmonary Fibrosis (an Update) and Progressive Pulmonary Fibrosis in Adults: An Official ATS/ERS/JRS/ALAT Clinical Practice Guideline. *Am J Respir Crit Care Med*. 2022;205(9):e18–e47.

8. Richeldi L, et al. Efficacy and Safety of Nintedanib in Idiopathic Pulmonary Fibrosis. *N Engl J Med*. 2014;370(22):2071–2082.

9. Flaherty KR, et al. Nintedanib in Progressive Fibrosing Interstitial Lung Diseases. *N Engl J Med*. 2019;381(18):1718–1727.

10. Schniering J, et al. Computed tomography-based radiomics decodes prognostic and molecular differences in interstitial lung disease related to systemic sclerosis. *Eur Respir J*. 2022;59(5). https://doi.org/10.1183/13993003.04503-2020.

11. Graham BL, et al. Standardization of Spirometry 2019 Update. An Official American Thoracic Society and European Respiratory Society Technical Statement. *Am J Respir Crit Care Med*. 2019;200(8):e70–e88.

12. Graham BL, et al. 2017 ERS/ATS standards for single-breath carbon monoxide uptake in the lung. *Eur Respir J*. 2017;49(1). https://doi.org/10.1183/13993003.00016-2016.

13. Holland AE, et al. An official European Respiratory Society/American Thoracic Society technical standard: field walking tests in chronic respiratory disease. *Eur Respir J*. 2014;44(6):1428–1446.

14. Wanger J, et al. Standardisation of the measurement of lung volumes. *Eur Respir J*. 2005;26(3):511–522.

15. Zwanenburg A, et al. The Image Biomarker Standardization Initiative: Standardized Quantitative Radiomics for High-Throughput Image-based Phenotyping. *Radiology*. 2020;295(2):328–338.

16. Irvin CG, Bates JHT. Measuring the lung function in the mouse: the challenge of size. *Respir Res*. 2003;4(1):4.

17. Rizzo S, et al. Radiomics: the facts and the challenges of image analysis. *Eur Radiol Exp*. 2018;2(1):36.

18. Aerts HJWL, et al. Decoding tumour phenotype by noninvasive imaging using a quantitative radiomics approach. *Nat Commun*. 2014;5:4006.

19. Aerts HJWL, et al. Defining a Radiomic Response Phenotype: A Pilot Study using targeted therapy in NSCLC. *Sci Rep*. 2016;6:33860.

20. Shrout PE, Fleiss JL. Intraclass correlations: uses in assessing rater reliability. *Psychol Bull*. 1979;86(2):420–428.

21. Koo TK, Li MY. A Guideline of Selecting and Reporting Intraclass Correlation Coefficients for Reliability Research. *J Chiropr Med*. 2016;15(2):155–163.

22. Hallal M, et al. Inference of kinase-signaling networks in human myeloid cell line models by Phosphoproteomics using kinase activity enrichment analysis (KAEA). *BMC Cancer*. 2021;21(1):789.

23. Yu F, et al. Fast Quantitative Analysis of timsTOF PASEF Data with MSFragger and IonQuant. *Mol Cell Proteomics*. 2020;19(9):1575–1585.

24. UniProt Consortium. UniProt: a worldwide hub of protein knowledge. *Nucleic Acids Res*. 2019;47(D1):D506–D515.

25. Huber W, et al. Variance stabilization applied to microarray data calibration and to the quantification of differential expression. *Bioinformatics*. 2002;18 Suppl 1:S96-104.

26. Uldry A-C, et al. Effect of Sample Transportation on the Proteome of Human Circulating Blood Extracellular Vesicles. *Int J Mol Sci*. 2022;23(9). https://doi.org/10.3390/ijms23094515.

27. Ritchie ME, et al. limma powers differential expression analyses for RNA-sequencing and microarray studies. *Nucleic Acids Res*. 2015;43(7):e47.

28. Schniering J, et al. 18F-AzaFol for Detection of Folate Receptor-β Positive Macrophages in Experimental Interstitial Lung Disease-A Proof-of-Concept Study. *Front Immunol*. 2019;10:2724.

29. Bankhead P, et al. QuPath: Open source software for digital pathology image analysis. *Sci Rep*. 2017;7(1):1–7.

30. Lawson RG, Jurs PC. New index for clustering tendency and its application to chemical problems. *J Chem Inf Comput Sci*. 1990;30(1):36–41.

31. Hennig C. Cluster-wise assessment of cluster stability. *Comput Stat Data Anal*. 2007;52(1):258–271.

32. Kuhn M. Building Predictive Models in R Using the caret Package. *J Stat Softw*. 2008;28:1–26.

33. Yu G, et al. clusterProfiler: an R package for comparing biological themes among gene clusters. *OMICS*. 2012;16(5):284–287.

34. Wu T, et al. clusterProfiler 4.0: A universal enrichment tool for interpreting omics data. *Innovation (Camb)*. 2021;2(3):100141.

35. Yu G, He Q-Y. ReactomePA: an R/Bioconductor package for reactome pathway analysis and

visualization. *Mol Biosyst*. 2016;12(2):477–479.

36. Mayr CH, et al. Integrative analysis of cell state changes in lung fibrosis with peripheral protein biomarkers. *EMBO Mol Med*. 2021;13(4):e12871.

37. Strunz M, et al. Alveolar regeneration through a Krt8+ transitional stem cell state that persists in human lung fibrosis. *Nat Commun*. 2020;11(1):3559.
